# Long Term Effects on Risk Factors for Cardiovascular Disease after 12-Months of Aerobic Exercise Intervention - A Worksite RCT among Cleaners

**DOI:** 10.1371/journal.pone.0158547

**Published:** 2016-08-11

**Authors:** Mette Korshøj, Mark Lidegaard, Peter Krustrup, Marie Birk Jørgensen, Karen Søgaard, Andreas Holtermann

**Affiliations:** 1 National Research Centre for the Working Environment, Lersø Parkallé 105, 2100, Copenhagen Ø, Denmark; 2 Department of Nutrition, Exercise and Sports, Copenhagen Centre for Team Sport and Health, University of Copenhagen, Nørre Allé 51, 2200, Copenhagen N, Denmark; 3 Sport and Health Sciences, College of Life and Environmental Sciences, St. Luke's Campus, University of Exeter, Exeter, United Kingdom; 4 Institute of Sports Science and Clinical Biomechanics, University of Southern Denmark, Campusvej 55, 5230, Odense M, Denmark; Kurume University School of Medicine, JAPAN

## Abstract

**Objectives:**

Occupational groups exposed to high occupational physical activity have an increased risk for cardiovascular disease (CVD). This may be explained by the high relative aerobic workload. Enhanced cardiorespiratory fitness reduces the relative aerobic workload. Thus, the aim was to evaluate the 12-months effects of worksite aerobic exercise on risk factors for CVD among cleaners.

**Methods:**

One hundred and sixteen cleaners aged 18–65 years were randomized to a group performing aerobic exercise and a reference group receiving lectures. Outcomes were collected at baseline and after 12-months. A repeated measures 2×2 multi-adjusted mixed-model design was applied to compare the between-group differences using intention-to-treat analysis.

**Results:**

Between-group differences (*p*<0.05) were found favouring the aerobic exercise group: cardiorespiratory fitness 2.15 (SE 1.03) mlO_2_/min/kg, aerobic workload -2.15 (SE 1.06) %HRR, resting HR -5.31 (SE 1.61) beats/min, high sensitive C-reactive protein -0.65 (SE 0.24) μg/ml. The blood pressure was unaltered. Stratified analyses on relative aerobic workload at baseline revealed that those with relative aerobic workloads ≥30% of HRR seems to impose a notable adverse effect on resting and ambulatory blood pressure.

**Conclusion:**

This long-term worksite aerobic exercise intervention among cleaners led to several beneficial effects, but also potential adverse effects among those with high relative aerobic workloads.

**Trial Registration:**

Controlled-Trials.com ISRCTN86682076

## Introduction

Individuals exposed to high occupational physical activity (OPA) suffer from an elevated incidence of cardiovascular disease (CVD) [1]. Cleaners are exposed to high OPA [2–5] consisting of working while being at one’s feet (standing, walking) [3–5] combined with frequent static working postures especially using the upper extremities (pushing/pulling the chart/mopping the floor) [3,4,6].

An explanation for the high incidence of CVD among cleaners [7,8] may be the combination of high OPA and a low cardiorespiratory fitness, generating a high relative aerobic workload [2–4,9,10]. A physical work task will expose a worker with a high cardiorespiratory fitness to a lower relative aerobic workload than a worker with a low cardiorespiratory fitness [9,11,12]. As cleaners generally have low cardiorespiratory fitness and high OPA [5,10,12], they are exposed to moderate to high aerobic workloads throughout the majority of their working time [2–5,10,12]. Long-term exposure of moderate to high relative aerobic workloads may lead to increased risk of CVD [13,14] due to the proposed harmful stress on the arterial endothelia [15].

Therefore, it is relevant to aim for a lowered risk for CVD among cleaners, i.e. by reducing the aerobic workload. A reduction of the aerobic workload may be achieved by an enhanced cardiorespiratory fitness [9,11,12]. Another benefit of an enhanced cardiorespiratory fitness is preservation of the cardiovascular health, as seen in general populations [16,17], but also among workers exposed to high OPA [18–21].

Recently, we performed a worksite intervention with aerobic exercise among cleaners. At 4-months follow-up the analysis showed that the cardiorespiratory fitness was enhanced, and the aerobic workload during cleaning, resting heart rate (RHR), sleeping heart rate (SHR) [10] and level of inflammation evaluated by high sensitive C-reactive protein (hsCRP) were reduced [22]. All of these results contribute to a lowering of the risk for CVD. However, also an increase in systolic blood pressure (BP) was found at 4-months follow-up [10,23]. This finding may be a significant adverse effect on CVD health, but also a transient physiological effect. Therefore, it is necessary to investigate the long-term effects from the intervention.

The main aim of this paper was therefore to evaluate the effects on risk factors for CVD from a cluster-randomized controlled trial among cleaners with worksite aerobic exercise after 12-months follow-up. We hypothesized that the worksite aerobic exercise intervention would enhance the cardiorespiratory fitness, reduce the aerobic workload, RHR, SHR, and level of inflammation evaluated by hsCRP. Additionally, the BP was hypothesised to be unaltered.

## Methods

### Study design

The study design has previously been described [24]. Briefly, the study was conducted as a cluster-randomized controlled trial with an aerobic exercise and a reference group. The intervention was divided into two phases with different aims. The first intervention phase, from baseline to 4-months follow-up, evaluated the efficacy of the intervention on risk factors for CVD [10,22,23]. The second intervention phase from 4-months to 12-months follow-up evaluated the long-term effects. Outcomes were measured at baseline, 4-months after baseline and 12-months after baseline [24]. The present paper reports the results from baseline to 12-months follow-up on risk factors for CVD.

The study was approved by the Danish data protection agency and the Ethics Committee for the regional capital in Denmark (journal number H-2-2011-116) by November 10^th^ 2011 and was conducted in accordance with the Helsinki declaration. The study was registered as ISRCTN86682076 in the Current Controlled Trials (http://www.controlled-trials.com/ISRCTN86682076). Before the etichs approval were obtained the preparation for the study, e.g. literature reviews for gaining knowledge of the latest validated monitors for diurnal measurements, were conducted.

### Recruitment of worksites and study participants

Cleaning companies in the suburban area of Copenhagen, Denmark, were recruited. Recruitment took place by directly telephoning or emailing the management of cleaning companies during the autumn of 2011, and was terminated by the last information meeting at May 15^th^ 2012. The recruitment of the companies and participants took place before the study was registred due to prolonged pilottesting of the diurnal measurements. If the management showed an interest in taking part in the project, a project meeting was arranged. If collaboration was confirmed, all employees were invited to an information meeting. The information meetings took place at the first company at November 22^nd^ 2011 and November 25^th^ 2011, at the second company at May 15^th^ 2012 and at the third company at June 22^nd^ 2012. At the information meeting the employed cleaners filled in a screening questionnaire, collecting background information such as ethnicity, smoking status and job seniority. Additionally, the cleaners were asked if they wanted to participate in the study.

At company level, inclusion criteria were: More than 50 employed cleaners and management’s permission for cleaners to participate in the project activities during paid working time. At participant level, inclusion criteria were: age between 18 and 65 years, employment as cleaning assistant for more than 20 hours per week, and signed informed consent to participate in the study. The only exclusion criterion for participating in the intervention was pregnancy. However, the following conditions excluded participation in the specific physical capacity tests: Congestive heart failure, hospital admission for myocardial infarction or acute coronary syndrome within the last two years, angina pectoris, severe hypertension (≥ 160 / ≥ 100 mmHg), serious or chronic illness, severe trauma, frequent migraine, and fever on the day of testing. Allergy to adhesive plasters excluded participation in diurnal heart rate (HR) measurements.

### Randomization

Randomization was performed at cluster-level, a cluster was set within strata, and stratums were formed according to which manager the participant reported to. Clusters were balanced on geographical work location, gender, age and job seniority. To minimize imbalance across several strata, the clusters were paired according to number of participants, gender, age and job seniority, within each stratum. The randomization was carried out by a researcher blinded to the identity of the participants, strata and clusters. The reliability of the randomization procedure was supervised by 3 researchers. All paired clusters assigned to the specific stratum were drawn from an opaque, tossed bag and was alternately allocated to either reference or aerobic exercise group, depending of the flip of a coin. Tails decided allocation of the first of the two drawn paired clusters to the reference group and heads to the aerobic exercise group. The second of the two drawn paired clusters was allocated to the opposite group to the first [24].

### Intervention

The authors confirm that all ongoing and related trials for this intervention are registrered. All project activities were carried out during paid working hours, at or near the worksite. The reference group was offered lectures, addressing healthy living, but not physical activity, and participants were invited to suggest topics. The aerobic exercise group were offered aerobic exercise sessions aiming to be performed at an intensity of ≥ 60% of maximal oxygen consumption (VO_2_max) [25]. Through a modified intervention mapping approach [26] the aerobic exercises were tailored to each of the enrolled companies [24]. The initial aerobic exercise activities offered were indoor biking, running on a treadmill and aerobics.

The intervention had a total duration of 12-months, divided in two intervention phases. Due to summer and Christmas holidays the activities were offered for a total of 42 weeks. During the first intervention phase the reference group received 2 lectures of 2 hours/lecture. The aerobic exercise group received supervised aerobic exercise of 2 x 30 minutes per week, corresponding to a total of 32 sessions (16 hours). During the second phase of the intervention, the reference group received 3 lectures of 2 hours/lecture while the aerobic exercise group received aerobic exercise of 2 x 30 min/week, in total 52 sessions (26 hours). The supervision of the aerobic exercise group was gradually declined, during the first 4-week period six sessions were supervised, during the second 4-week period five sessions were supervised, during the third 4-week period four sessions were supervised, during the fourth 4-week period two sessions were supervised and during the fifth and sixth 4-week period only one session was supervised per period. Participation in the aerobic exercise group was registered only when the instructor was present.

### Data collection

All participants were tested at baseline and after 4 and 12-months intervention period at the worksite during paid working hours. The baseline measurements were conducted in January 2012 at the first company, in May 2012 at the second company and in August 2012 at the third company, 4-months follow-up measurements were conducted in May/June 2012 at the first company, in January/February 2013 at the second company and in February 2013 at the third company, 12-months follow-up measurements were conducted in January 2013 at the first company, in June 2013 at the second company and in September 2013 at the thierd company. The test consisted of a structured interview, physical testing of health- and capacity-related measures and objective diurnal measures of HR and ambulatory blood pressure (ABP) [24]. Participants got instant feedback from the physical testing and were encouraged to contact a physician if their systolic or diastolic BP exceeded recommended levels (≥ 140 or ≥ 90 mmHg) [27].

The interview assessed gender, ethnicity, country of birth, education, occupational group, employment status, job seniority, occupational and leisure time physical activity [28], smoking, general health [29], diagnosed illnesses, daily use of heart, anti-hypertensive, and cholesterol reducing medication. Ethnicity was classified as western or non-western based on country of birth. All European countries, Australia, Canada, and USA were considered western.

Physical examinations measured body weight (kg) and body fat (%) with bio-electric-impedance-analysis tool Tanita BC418, height with Seca model 213 1721009, waist circumference with Seca model 201. The waist was defined as the narrowest point between the lowest rib and the iliac crest [30,31]. Body mass index (BMI), was calculated as body weight (kg) divided by body height squared (m^2^) [30,31]. Blood pressure was measured with Omron M6 Comfort on the upper left arm after minimum 15 minutes of sitting rest. Measurements were conducted with participants wearing light clothes and no shoes. Level of cardiorespiratory fitness was estimated by a sub-maximal step test [32] conducted on a bench of 30 cm height for females and 35 cm for males. Step frequency was increased from 0.2 steps per second to maximal 0.8 steps per second, at maximal 6 minutes of testing time. The step test was terminated when the participant could no longer keep the stepping rhythm or properly extend the knee.

The diurnal measurements of HR were performed with Actiheart (www.camntech.com). Actiheart is validated for measurement of HR, HR variability and estimation of energy expenditure in the field [33–35]. The electrocardiographic raw signals are measured with a sensitivity of 0.25 mV and HR is calculated as the number of R peaks in the QRS complex per minute. Before measurement, the Actiheart monitor was initialised by gender and age, and mounted with ag-ag-cl pre-gelled electrodes (Ambu blue sensor VL-00-S/25) at one of the validated body positions [36]. Heart rate was measured over four days (mostly 2 working and 2 non-working days). Participants were instructed in how to wear the monitor and to write a log of working hours, sleeping and waking time and time periods spent without monitors. During the diurnal measurements, participants were asked to live their normal every-day life.

Number of steps taken were measured with the Actigraph GT3X + (www.theactigraphcorp.com) and analyzed in the customized software Acti4 [37,38]. The Actigraph was mounted on the skin with adhesive tape (3 M, Hair-Set, double sided adhesive tape and Fixomull, BSN medical), on the right thigh medial to the iliac crest and the top of the patella. The Actigraph was orientated with the x-axis pointing downwards, y-axis horizontally to the left and z-axis horizontally forward.

Ambulatory blood pressure (ABP) measurements were performed with Spacelabs 90217 (www.spacelabshealthcare.com) [39], by oscillometry, mounted on the non-dominant upper arm with a tube connecting the sampler to the cuff. The sampler was mounted with elastic straps around the waist, and the frequency of measurements was every 20 minutes during waking hours, and every 40 minutes during sleep [40,41]. The participants were instructed to keep quiet and keep the arm at rest while the measurement was proceeding. If a measurement failed, the monitor automatically measured again a few minutes later. Participants were asked to live their normal every-day life and they were instructed in taking on and off the Spacelabs when showering.

The participants were instructed to write a log of working hours, sleeping and waking time and time periods spent without monitors. This enabled a classification of the 24 hours into work/leisure/sleep hours during data analysis.

### Blood sampling

A 25-ml non-fasting blood sample was taken from the vena brachialis by trained laboratory technicians. The samples were taken during working hours (7 a.m. to 3 p.m.) and no restrictions were imposed with regard to food, caffeine, tobacco and alcohol consumption or exercise prior to the sampling. The samples were stored at -20°C and analyzed within a maximum of 2 years for high density lipoprotein (HDL) cholesterol, low density density lipoprotein (LDL) cholesterol and total cholesterol (TC), triacylglycerol (TG) and glycated hemaglobin (HbA_1c_). Samples of ethylenediaminetetraacetic acid (EDTA) plasma were stored at -80°C until analyzed for hsCRP and fibrinogen within a maximum of 2 years.

A high performance liquid chromatography (HPLC) method was used for determination of HbA_1c_. The HPLC consisted of a Waters 625 LC system together with a Waters photodiode array detector model 996 and a WISP 717 auto sampler for automatic injection of the samples. Millennium chromatography software was used to calculate concentrations (Waters Associates Inc., Milford, US). A cation exchange column Mono S HR 5/5 from Pharmacia Biotech AB, Uppsala, Sweden was used to separate HbA_1c_ from other components in the samples. The method for HbA_1c_ has been evaluated by inter-laboratory comparison based on 17 patient samples and found to be linear in the range 4.1‒14.3% of total haemoglobin and without systematic bias [42]. Lyphochek Diabetes Control (Calibrator) from BioRad (Anaheim, CA, US) for HbA_1c_ was used to monitor the long-term stability of the method.

HDL cholesterol, LDL cholesterol and TC were analyzed using a Cobas MIRA Plus. The determination of HDL cholesterol, LDL cholesterol, TC and TG was carried out by ABX Pentra assays from Triolab (Sollentuna, Sweden). Calculation of LDL/HDL cholesterol ratio and LDL/TC cholesterol ratio was carried out by dividing LDL cholesterol by HDL cholesterol and LDL cholesterol by TC. The analytical methods for measuring TC in serum have been evaluated by a method evaluation function design [43] to estimate the random and systematic effects. This was based on a linear least squares regression analysis of the measured concentration vs the conventional true concentration of a series of method evaluation samples containing cholesterol. The between-assay variation was estimated to be 2.7% at 5.3 mmol/L cholesterol [44]. Commercially available control samples for HDL cholesterol, LDL cholesterol, TC and TG were analyzed together with the samples to show equivalence between different runs. Westgard control charts were used to document that the analytical methods remained in analytical and statistical control, i.e. that the precision and trueness of the analytical methods remained stable [45].

Fibrinogen was analyzed by turbidimetry on Cobas MIRA Plus. We used a high sensitive ELISA (EU59151), purchased from IBL, International GMBH, Hamburg, Germany, to measure hsCRP. The between-assay variation was 5.8% at 1.6 μg/ml and the limit of detection was 0.02 μg/ml.

### Sample Size

The power calculation, performed prior to the study showed that an expected increase in cardiorespiratory fitness of 4% would need 52 participants in each of the two intervention groups to show significance at a level of 0.05. Sample size calculations assumed recruitment of 40% of the eligible cleaners and a dropout rate of 30% during the intervention [24].

### Analyses

The primary outcome in the 12-month study is change in cardiorespiratory fitness (mlO_2_∙min^-1^∙kg^-1^) from baseline to 12-month follow-up. Secondary outcomes are changes in relative aerobic workload (% HRR), RHR in beats-per-minute (bpm), SHR (bpm), resting systolic and diastolic BP, 24 hour ambulatory BP (ABP), and level of biomarkers related to risk for CVD (e.g. concentration of high sensitive C-reactive protein).

Only HR measurements with beat error of ≤ 50% were included to meet the data quality criteria set by Skotte and colleagues [37]. The HR reserve (HRR) was calculated as the difference between the estimated maximal HR (HR_max_), estimated by (208–0.7 * age) [46], and the resting HR (RHR) (HRR = HR_max_−RHR) [9]. The RHR was defined as the 10^th^ lowest recorded HR value during 24 hours [47]. The relative aerobic workload (% HRR) was calculated as the observed HR percentage of the HRR ((observed HR–RHR) * 100% / HRR) [9].

ABP measurements were included when a minimum of 25% of planned measurements were complete [39,40]. All measures of ABP were visually checked for physiological outliers (systolic BP < 80 and > 240 mmHg, diastolic BP < 50 and > 130 mmHg). For the ABP analysis, the HR measurements and estimated aerobic workloads were analysed in 5 minutes intervals preceding each ABP measurement.

### Statistical analysis

Statistical analyses were performed using the IBM SPSS statistics software (version 21) (Armonk, NY, US) and the SAS statistical software for Windows (version 9.3) (Cary, NC, US).

All analyses were performed according to the intention to treat principle (ITT), in which all randomized participants are included in the statistical analyses [48]. Missing values were not imputed neither for outcome nor covariate variables [49]. Both within and between-groups 12-month changes of all outcomes were computed with standard errors and 95% confidence intervals. Differences in 12-month changes of all outcomes were analyzed in a repeated-measures 2 x 2 mixed models design. Independent categorical variables (fixed factors) were group (aerobic exercise and reference), measurement time (baseline and 12-months follow-up), and the interaction between group and measurement time. Participants were entered in the model as a random effect nested in clusters to account for the cluster-based randomization. Covariates were chosen based on baseline differences between groups on theoretically considered confounders and their statistical association with the group and measurement time.

For the primary outcome, and the secondary outcomes on aerobic workload, RHR, SHR and resting BP, the following covariates (reference value in parenthesis) were entered into the mixed models in the following incremental steps: 1) baseline value of the respective outcome, 2) age, gender (male), daily use of antihypertension and/or heart medication (none), smoking status (never smoking and/or currently non-smoking), and either level of leisure time physical activity (< 2 h/weeks light activity) or baseline cardiorespiratory fitness. The intervention effect estimates were reported as between group mean difference ± standard error, 95% confidence interval, and p-value.

For the secondary outcomes on 24 hour ABP, the following covariates were stepwise included in the analysis (reference value in parenthesis): model 1: mean baseline ABP from the respective time period; model 2: additionally age, gender (male), daily use of hypertension and / or heart medication (none), smoking (never smoking and/or currently non-smoking), and cardiorespiratory fitness at baseline (high). The estimated cardiorespiratory fitness was coded as a categorical variable split in tertiles (low, medium and high), and those participants who did not conduct the cardiorespiratory fitness test constituted an extra category.

For the secondary outcomes on biomarkers related to risk for CVD, the following covariates were incrementally taken into account in the analysis (reference value in parenthesis): model 1: baseline value of the respective outcome; model 2: additionally age, gender (male), BMI, daily use of cholesterol and or hormone medication (none), smoking status (never smoked and/or currently non-smoking), level of leisure time physical activity (< 2 hours per weeks light activity) and alcohol consumption.

For the secondary outcomes on body weight, fat percentage and waist circumference, the following covariates were incrementally taken into account in the analysis (reference value in parenthesis): model 1: baseline value of the respective outcome; model 2: additionally age, gender (male), BMI, smoking status (never smoked and/or currently non-smoking), level of leisure time physical activity (< 2 hours per weeks light activity) and alcohol consumption.

Secondarily, a sensitivity analysis excluding participants reporting use of antihypertension and/or heart medication on a daily basis was performed for the outcomes on cardiorespiratory fitness, RHR, SHR, aerobic workload, resting and 24h systolic and diastolic blood pressure, using the same fully adjusted statistical model.

Additionally, sensitivity analysis was performed by applying the same fully adjusted statistical model on the outcomes of hsCRP, fibrinogen, HDL, LDL, TC, TG and HbA1c with the exclusion of participants reporting daily use of cholesterol and/or hormone medication.

A secondary between-group analysis stratified on baseline level of aerobic workload (low <30% HRR or high ≥30% HRR) was conducted. The cutpoint of high and low aerobic workload were based on previous studies [50,51].

## Results

### Flow of participants

**Fig 1:**
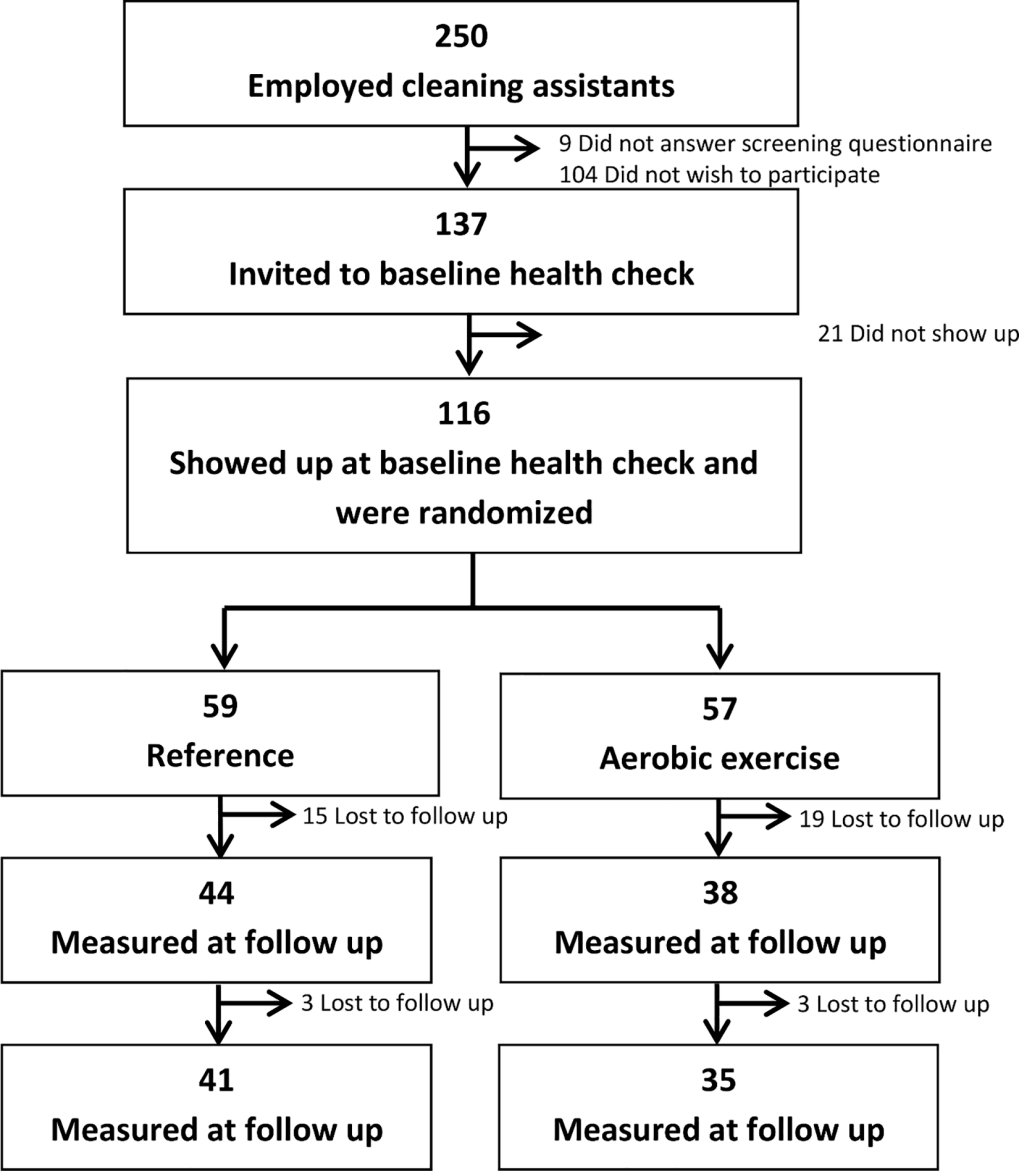
Flow of the participants.

All three contacted companies agreed to participate. The study was presented to 250 cleaning assistants at these companies. Of those, 137 (45%) consented to participate (consenters) and were invited to the baseline measurement. Baseline measurements were conducted among 116 of them and they were randomized, with 59 assigned to the reference group and 57 to the aerobic exercise group. After baseline measurements, 42 (36%) participants (18 from the reference and 24 from the aerobic exercise group) dropped out of the study and were lost to follow-up at 12-months (Fig 1).

The enrolled cleaners cleaned offices, hospitals, kindergartens, schools and sports facilities. Occupational physical activities among this population of cleaners were determined by the work plans and requirements made by their managers. The work plans described which rooms should be cleaned during the week, the quality of cleaning of different surfaces and areas, empting of garbage bins and wash of curtains, bed linen etc. The majority of the occupational physical activities took place while the cleaner was standing or walking sometimes while the back is forward/sideways bended, some took place while the cleaner was squatting, kneeling, sitting or standing on all four and some took place with one or both arms lifted above shoulder height.

The following significant (*p* < 0.05) differences were seen between the consenters and non-consenters: the consenters had lower job seniority, were more frequently born outside Denmark, and had suffered more musculoskeletal disorders in the shoulders, arms/wrists and feet/ankles within the last 30 days. No differences were seen in diagnosed illnesses, level of OPA and LTPA, gender, age, height, weight and job seniority.

The main self-reported reasons for non-participation among non-consenters were lack of time (40%) and not finding the study relevant (11%) [10]. Of the consenters, 21 were not randomized because they withdrew from the study before the baseline measurement took place, or did not show up at the baseline measurement. The following significant (*p* < 0.05) differences were seen between those consenters who were randomized and the consenters not being randomized: the consenters being randomized were more frequently born outside Denmark.

### Compliance

The 36% drop out at 12-months follow-up was above the expected 30% in the power calculations.

Adherence during the implementation period was not registered because not all sessions were supervised. The drop out at 4-months follow-up was within the expected in the power calculations [10]. After every fourth week of the 4-month intervention, the HR was monitored during the aerobic exercise session yielding an average HRR of 67% [standard deviation (SD) 13]. Overall, 94% of the planned sessions were offered as planned.

### Baseline characteristics of the study population

The baseline characteristics of the study population are presented in Table 1. Participants randomized to the intervention were mainly immigrants; 86% stated to have another place of birth than Denmark, of those 62% were non-western. Within the aerobic exercise group, those lost to follow-up (n = 24) had a significantly shorter job seniority (5 years), and significantly more stated to be current smokers (25%). There was a tendency (*p* = 0.053) towards that more stated their OPA as strenuous (29%). Within the reference group those lost to follow-up (n = 16) had a significantly higher RHR (5.4 bpm).

**Table 1:** Description of the randomized study population of cleaners at baseline (N = 116), stratified by the intervention group.

	Randomized population (N = 116)			Aerobic exercise (n = 57)			Reference(n = 59)		
	Mean	SD	N	Mean	SD	n	Mean	SD	N
Age (years)	45.3	8.6		44.9	9.2		45.7	8.1	
Gender (% females)	75.9		88	75.4		43	76.3		45
Height (m)	1.6	0.09		1.6	0.09		1.6	0.08	
Body weight (kg)	70.7	14.1		69.7	12.7		71.7	15.4	
BMI (kg/m^2^)	26.7	4.5		26.2	4.0		27.1	4.9	
Body fat (%)	31.6	8.6		31.1	8.3		32.1	8.9	
Waist circumference (cm)	87.6	11.1		86.7	11.0		88.4	11.2	
Job seniority (years)	11.9	7.8		12.3	8.7		11.5	6.8	
Current smoker (%)	24.1		28	22.8		13	25.4		15
Ethnicity (% non western)	62.1		72	70.2		40	54.2		32
Daily use of antihypertension and/or heart medication (%)	12.1		14	14.0		8	10.2		6
Daily use of cholesterol and/or hormone medication (%)	13.8		16	12.3		7	15.3		9
Leisure time physical activity (% < 2 h/weeks light activity or light activity 2–4 h/weeks)	72.4		84	78.9		45	66.1		39
Physical activity at work (% having standing/walking work including lifts and strenuous physical work)	60.3		70	63.2		36	57.6		34
Resting systolic blood pressure (mmHg)	122.7	21.7		125.2	25.1		120.3	17.5	
Resting diastolic blood pressure (mmHg)	82.7	12.6		83.7	14.2		81.7	10.8	
Cardiorespiratory fitness (mlO_2_∙min^-1^∙kg^-1^)	24.9	6.6		24.8	5.8		25.0	7.2	
Resting heart rate (beat/min)	71.3	14.8		71.7	10.6		70.5	8.8	
Sleeping heart rate (beat/min)	49.5	5.8		49.2	6.5		49.7	5.1	
Aerobic work load during work (% of HRR)	30.9	7.2		30.1	6.7		31.7	7.5	
Ambulatory systolic blood pressure (mmHg)	120.6	12.7	83	121.8	14.7	41	119.5	10.4	42
Ambulatory diastolic blood pressure (mmHg)	77.3	7.7	83	77.3	8.1	41	77.3	7.5	42
hsCRP (μg/ml)	1.47	1.72		1.39	1.30		1.53	2.05	
Fibrinogen (g/l)	3.22	0.68		3.24	0.69		3.19	0.67	
HDL (mmol/L)	1.53	0.39		1.53	0.36		1.53	0.41	
LDL (mmol/L)	3.32	1.00		3.50	0.98		3.16	1.00	
TC (mmol/L)	5.66	1.25		5.82	1.25		5.50	1.24	
TG (mmol/L)	1.45	0.77		1.48	0.82		1.42	0.71	
HbA1c (%)	5.24	0.65		5.29	0.77		5.20	0.51	

Note: Mean ± standard deviations or percent (n) are reported. [SD = standard deviation; HRR = heart rate reserve]. Differences between aerobic exercise and reference groups were analyzed with student’s t-test for continuous variables, and with Chi^2^ test for categorical variables.

### Intervention effects

The between-groups differences in 12-month change of primary and secondary outcomes are reported in Table 2 for the fully-adjusted model 2.

**Table 2:** Within groups means pre and post intervention, between groups difference (mean ± standard error) from baseline to 12-months follow-up and percentage change relative to the baseline mean of the randomized population of cleaners (N = 116).

	Aerobic exercise		Reference							
	Pre	Post	Pre	Post	Δ	%	SE	95% CI	*p*	n
Fitness^a^ (mlO_2_/min/kg)	24.25	28.31*	24.07	26.16*	2.15	8.63	1.03	0.11 to 4.19	0.04	80
Resting heart rate^b^ (beat/min)	70.68	68.73	70.68	74.04*	-5.31	7.45	1.61	-8.50 to -2.12	<0.01	81
Sleeping heart rate^b^ (beat/min)	50.72	50.75	50.50	52.11	-1.37	2.77	1.09	-3.54 to 0.81	0.22	67
Aerobic workload^b^ (% HRR)	29.47	27.38*	29.71	29.53	-2.15	6.96	1.06	-4.28 to -0.03	<0.05	60
Resting systolic blood pressure^b^ (mmHg)	117.22	116.48	117.42	112.66*	3.82	3.11	2.38	-0.90 to 8.53	0.11	81
Resting diastolic blood pressure^b^ (mmHg)	79.87	75.56*	79.96	77.07*	-1.51	1.83	1.34	-4.15 to 1.14	0.26	81
24h ambulatory systolic blood^c^ pressure (mmHg)	118.74	121.44*	119.71	119.91	1.53	1.27	1.52	-1.61 to 4.66	0.33	30^e^
24h ambulatory diastolic blood^c^ pressure (mmHg)	76.67	77.03	76.36	76.95	0.08	0.10	1.05	-2.08 to 2.25	0.94	30^e^
Δ hsCRP^d^ (μg/ml)	1.64	1.41	1.63	2.06*	-0.65	44.22	0.24	-1.12 to -0.19	<0.01	87
Δ Fibrinogen^d^ (g/l)	3.29	3.45	3.29	3.49*	-0.03	0.93	0.09	-0.21 to 0.15	0.71	87
Δ HDL^d^ (mmol/L)	1.55	1.50	1.56	1.51	-0.005	0.33	0.04	-0.09 to 0.08	0.90	87
Δ LDL^d^ (mmol/L)	3.57	2.71*	3.58	2.93*	-0.23	6.93	0.13	-0.48 to 0.02	0.07	87
Δ TC^d^ (mmol/L)	5.90	5.60*	5.95	5.92	-0.32	5.65	0.14	-0.60 to -0.04	0.02	87
Δ TG^d^ (mmol/L)	1.46	1.61	1.48	1.68	-0.08	5.52	0.13	-0.33 to 0.18	0.57	87
Δ HbA1c^d^ (%)	5.30	5.39	5.32	5.36	0.03	5.25	0.06	-0.09 to 0.15	0.66	87
Body weight^f^ (kg)	71.98	71.66	71.93	73.31*	-1.65	0.65	0.92	-2.94 to -0.36	0.01	95
Body fat^f^ (%)	31.88	31.01	31.73	32.45	-1.45	0.50	1.58	-2.43 to -0.47	<0.01	95
Waist circumference^f^ (cm)	89.24	86.32*	89.13	88.24	-1.92	0.92	1.05	-3.74 to -0.11	0.04	95

Note: Between group 95% confidence interval and level of significance are reported. The n differs between the different outcomes due to missing observations in covariate and/or outcome variables.

All outcomes are adjusted for baseline value of the respective outcome, age, gender (male), smoking status (never smoking and/or currently non-smoking), * within group difference (p < 0.05),

^a^ additionally adjusted for daily use of antihypertension and/or heart medication (none), and baseline cardiorespiratory fitness

^b^ additionally adjusted for daily use of antihypertension and/or heart medication (none), and level of leisure time physical activity (< 2 hours per weeks light activity)

^c^ additionally adjusted for daily use of antihypertension and/or heart medication (none), unequal amount of ambulatory blood pressure measurements during work/leisure/sleep and baseline cardiorespiratory fitness in tertiles (high)

^d^ additionally adjusted for BMI, daily use of cholesterol and or hormone medication (none), level of leisure time physical activity (< 2 hours per weeks light activity) and alcohol consumption

^e^ of the 30 included participants 17 were randomized to the aerobic exercise group and 13 were randomized to the reference group

^f^ additionally adjusted for age, gender (male), BMI, smoking status (never smoked and/or currently non-smoking), level of leisure time physical activity (< 2 hours per weeks light activity) and alcohol consumption.

**Fig 2:**
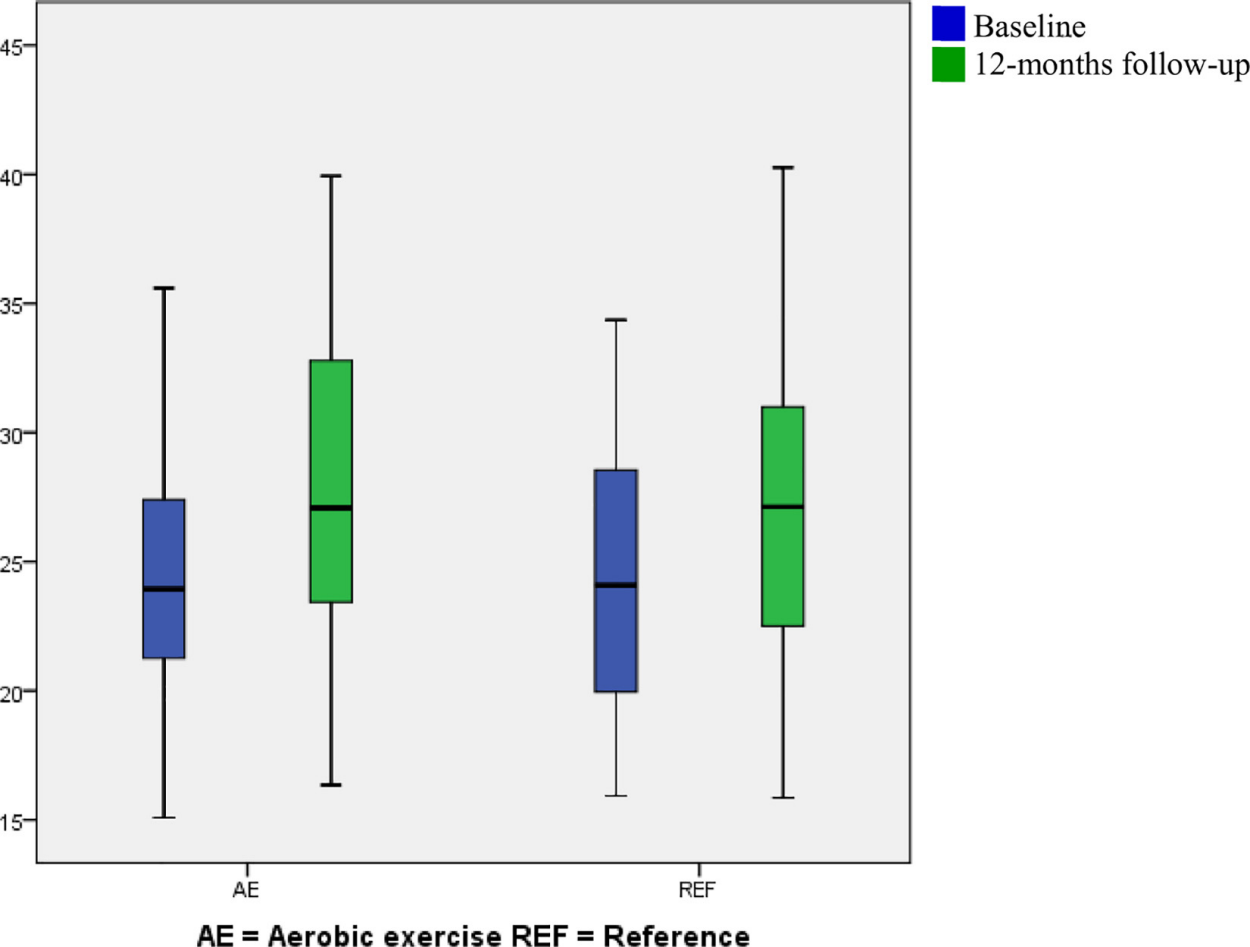
Box plot (mean, SD and range) of the cardiorespiratory fitness divided by intervention group at baseline and 12-months of follow up. Blue represents baseline and green represents 12-months follow-up.

**Fig 3:**
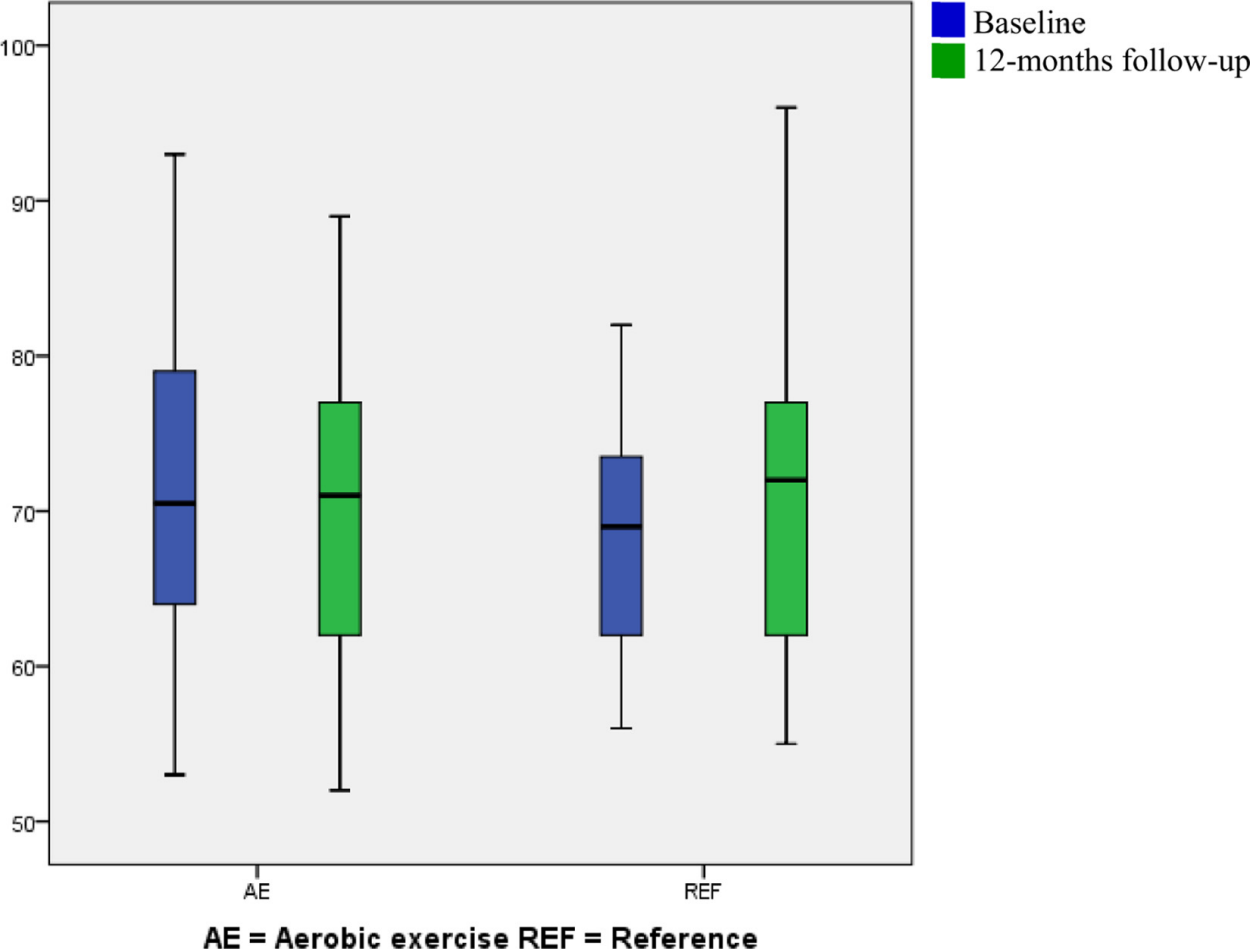
Box plot (mean, SD and range) of the resting heart rate divided by intervention group at baseline and 12-months of follow up. Blue represents baseline and green represents 12-months follow-up.

The fully adjusted between-groups difference in cardiorespiratory fitness change was 2.15 ± 1.03 mlO_2_/min/kg (95% CI 0.11 to 4.19, *p* 0.04) in the aerobic exercise group relative to the reference group (Fig 2). This corresponds to an 8.6% increase relative to the overall baseline mean in the randomized population. Significant between-groups difference in change of RHR was -5.31 ± 1.61 bpm (95% CI -8.50 to -2.12, *p* <0.01) (Fig 3). Relative to the overall baseline mean in the randomized population, this decrease corresponds to 7.5%. An unaltered SHR was seen in the fully-adjusted between-groups analysis. Also for aerobic workload significant between-groups differences were found; -2.15 ± 1.06% HRR (95% CI -4.28 to 0.03, *p* <0.05). This corresponds to a 7.0% decrease relative to the overall baseline mean in the randomized population. The change in resting systolic BP showed a tendency towards a between-groups differences of 3.82 ± 2.38 mmHg (95% CI -0.90 to 8.53, *p* 0.11). Resting diastolic BP changes did not differ significantly between groups. No changes were seen in either systolic or diastolic ABP.

Significant between-groups difference in change of hsCRP was -0.65 ± 0.24 μg/ml (95% CI -1.12 to -0.19, *p* <0.01). A tendency towards a between-group difference in LDL cholesterol was seen -0.23 ± 0.13 mmol/L (95% CI -0.48 to 0.02, *p* 0.07). Significant between-groups difference in change of TC was -0.32 ± 0.14 mmol/L (95% CI -0.60 to -0.04, *p* 0.02). Non-significant between-group changes in fibrinogen, HDL cholesterol, TG and HbA1c were found in the fully-adjusted model.

**Fig 4:**
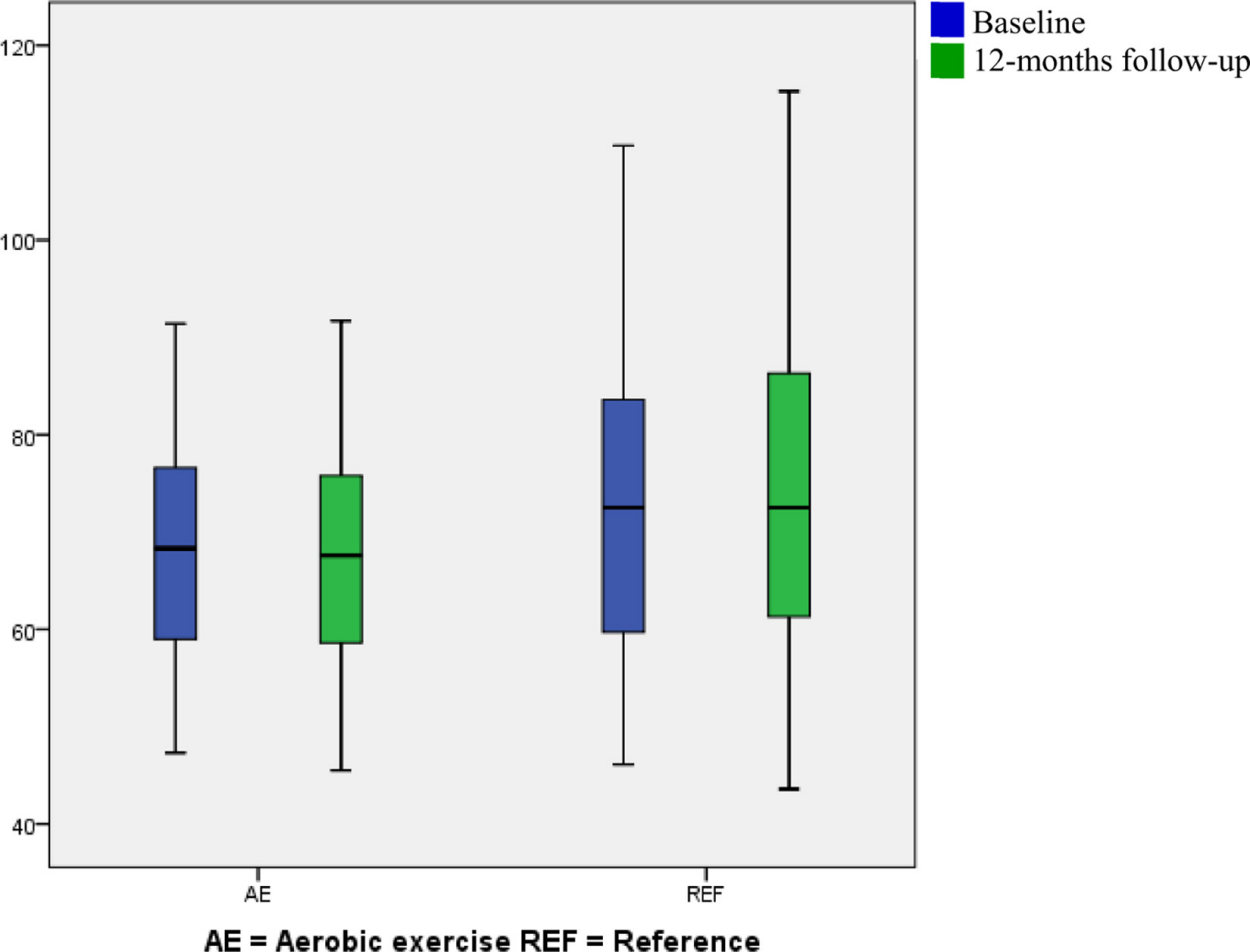
Box plot (mean, SD and range) of the body weight divided by intervention group at baseline and 12-months of follow up. Blue represents baseline and green represents 12-months follow-up.

Significant between-groups difference in reductions of body weight (-1.65 ± 0.92 kg, 95% CI -2.94 to -0.36, *p* 0.01) (Fig 4), body fat (-1.45 ± 1.58%, 95% CI -2.43 to -0.47, *p* <0.01) and waist circumference (-1.92 ± 1.05 kg, 95% CI -3.74 to -0.11, *p* 0.04) were seen.

### Sensitivity analysis

For the majority of the outcomes, the results from the unadjusted and the fully adjusted models are numerically and statistically similar. Only the numerical results regarding SHR, 24 hour systolic ABP, HDL and TG changes from a positive change estimate (unadjusted model) to a negative change estimate (fully adjusted model), however these dissimilarities do not affect the 95% CI much, thereby not changing the conclusions.

Sensitivity analysis excluding the 14 participants ingesting antihypertensive and heart medication yielded between-groups differences during follow-up that were comparable to the majority of the results in the entire randomized population (N = 116), but not reaching statistical significance on the change in cardiorespiratory fitness (*p* 0.34) and with a statistical decrease in resting diastolic blood pressure of -2.93 ± 1.38 mmHg (95% CI -5.65 to -0.20, *p* 0.04). Additionally, in the sensitivity analysis excluding participants reporting a daily use of cholesterol and/or hormone medication (n = 16), the analysis yielded numerically and statistically similar between-group differences from baseline to follow-up in the fully adjusted model.

### Stratified analysis

An analysis stratified on baseline level of aerobic workload (≥ or <30% HRR) of between groups difference (mean ± standard error) from baseline to 12-months follow-up of primary and secondary outcomes are reported in Table 3 for the fully-adjusted model 2. Overall these analyses lack statistical power, however the participants exposed to an aerobic workload ≥30% HRR at baseline seems to obtain adverse effects related to an increased overall risk of cardiovascular disease.

**Table 3:** Between groups means pre and post intervention stratified on baseline level of aerobic workload (≥ or <30% HRR), between groups difference (mean ± standard error) from baseline to 12-months follow-up (N = 116).

	Aerobic workload ≥30% HRR					Aerobic workload <30% HRR				
	Δ	SE	95% CI	*P*	n	Δ	SE	95% CI	*p*	n
Fitness^a^ (mlO_2_/min/kg)	-0.67	1.62	-3.96 to 2.62	0.68	28	1.73	2.29	-2.92 to 6.38	0.46	31
Resting heart rate^b^ (beat/min)	0.03	2.31	-4.64 to 4.70	0.99	29	-6.60	2.76	-12.18 to -1.02	0.02	30
Sleeping heart rate^b^ (beat/min)	-0.71	1.29	-3.34 to 1.92	0.59	29	-0.84	2.45	-5.84 to 4.16	0.73	31
Aerobic workload^b^ (% HRR)	-1.61	1.61	-4.90 to 1.68	0.33	29	-3.04	1.51	-6.12 to 0.04	0.05	31
Resting systolic blood pressure^b^ (mmHg)	9.95	5.46	-1.09 to 20.99	0.08	29	-3.31	3.36	-10.11 to 3.48	0.33	30
Resting diastolic blood pressure^b^ (mmHg)	3.36	2.62	-1.93 to 8.65	0.21	29	-2.79	2.12	-7.08 to 1.51	0.20	30
24h ambulatory systolic blood^c^ pressure (mmHg)	6.46	4.37	-7.16 to 20.08	0.23	13	-3.35	2.95	-11.54 to 4.83	0.32	11
24h ambulatory diastolic blood^c^ pressure (mmHg)	5.74	6.12	-13.47 to 24.95	0.42	13	-6.33	1.46	-9.86 to -2.79	<0.01	11
Δ hsCRP^d^ (μg/ml)	-0.73	0.61	-1.95 to 0.50	0.24	32	-0.10	0.28	-0.67 to 0.47	0.72	32
Δ Fibrinogen^d^ (g/l)	0.13	0.17	-0.22 to 0.47	0.46	32	-0.07	0.14	-0.34 to 0.21	0.61	32
Δ HDL^d^ (mmol/L)	0.01	0.11	-0.21 to 0.23	0.92	32	0.06	0.05	-0.04 to 0.16	0.22	32
Δ LDL^d^ (mmol/L)	-0.14	0.28	-0.71 to 0.43	0.63	32	-0.14	0.22	-0.58 to 0.30	0.52	32
Δ TC^d^ (mmol/L)	0.30	0.31	-0.33 to 0.93	0.34	32	-0.70	0.22	-1.14 to -0.25	<0.01	32
Δ TG^d^ (mmol/L)	0.31	0.23	-0.15 to 0.77	0.19	32	-0.17	0.27	-0.72 to 0.38	0.53	32
Δ HbA1c^d^ (%)	0.12	0.11	-0.10 to 0.34	0.28	32	0.09	0.11	-0.12 to 0.31	0.38	32
Body weight^e^ (kg)	-1.47	1.41	-4.31 to 1.38	0.30	35	-1.75	0.67	-3.10 to -0.41	0.01	34
Body fat^e^ (%)	-0.99	0.90	-2.79 to 0.82	0.28	35	-0.84	0.57	-1.99 to 0.32	0.15	34
Waist circumference^e^ (cm)	1.11	1.69	-2.29 to 4.51	0.51	35	-4.86	1.65	-8.19 to -1.54	<0.01	34

Note: Between group 95% confidence interval and level of significance are reported. The n differs between the different outcomes due to missing observations in covariate and/or outcome variables.

All outcomes are adjusted for baseline value of the respective outcome, age, gender (male), smoking status (never smoking and/or currently non-smoking),

^a^ additionally adjusted for daily use of antihypertension and/or heart medication (none), and baseline cardiorespiratory fitness

^b^ additionally adjusted for daily use of antihypertension and/or heart medication (none), and level of leisure time physical activity (< 2 hours per weeks light activity)

^c^ additionally adjusted for daily use of antihypertension and/or heart medication (none), and baseline cardiorespiratory fitness in tertiles (high)

^d^ additionally adjusted for BMI, daily use of cholesterol and or hormone medication (none), level of leisure time physical activity (< 2 hours per weeks light activity) and alcohol consumption.

^e^ additionally adjusted for age, gender (male), BMI, smoking status (never smoked and/or currently non-smoking), level of leisure time physical activity (< 2 hours per weeks light activity) and alcohol consumption.

## Discussion

The main results of this study among cleaners are that the aerobic exercise group significantly enhanced cardiorespiratory fitness, reduced RHR, aerobic workload, hsCRP, body weight, body fat and waist circumference compared to the reference group.

Results from 4-months of follow-up have earlier been reported from this worksite intervention [10,22]. However, the unexpected finding of an adverse effect of the aerobic exercise on the resting blood pressure [10] reports from a longer follow-up is warranted. Additionally, this worksite intervention was implemented during the working hours and therefore could have induced a behavioral change causing sustained effects on health; consequently this paper includes data on body composition.

### Primary outcome

Between-group comparisons including all randomized participants show an average enhanced cardiorespiratory fitness of 8.63%, which is in accordance with findings from earlier worksite physical exercise interventions [52–55]. Likewise, this enhanced cardiorespiratory fitness at 12-months is in accordance with the result from 4-months follow-up [10]. More than 15% of all-cause mortality among a population of women is expected to be attributed to low cardiorespiratory fitness [16]. An increased cardiorespiratory fitness by 3.5 mlO_2_/min/kg is estimated to reduce the incidence of cardiovascular mortality with 15% [56]. Thus, an enhanced cardiorespiratory fitness of the magnitude reported in this study potentially lowers the overall risk of CVD [16,56].

To enhance cardiorespiratory fitness, an exercise intensity of ≥60% of HRR is required [25]. During the initial 4-months of the aerobic exercise sessions, the average HRR during the aerobic exercise sessions in this study was 67% (SD 13). After the initial 4-months of the aerobic exercise sessions the intensity was not collected. However, the increased cardiorespiratory fitness after 12-months indicates that the participants remained to have an adequate intensity HRR throughout the intervention and participating in aerobic exercise 2x30 min/week.

### Secondary outcomes

The aerobic exercise group significantly reduced their RHR (n = 81) by 7.45% compared to the reference group, while the SHR (n = 67) did not significantly differ between the groups, possibly due to the small sample size. The reduced RHR may be explained by the enhanced level of cardiorespiratory fitness implying an increased capacity as well as effectiveness in distribution and metabolism of oxygen, such as an increased stroke and minute volume [57]. Reductions in RHR and SHR have been related to a reduced risk of CVD [58,59]. Likewise, a graded increased risk of CVD has been shown in relation to increasing RHR in both healthy men and women [58].

The aerobic exercise group significantly reduced their aerobic workload by 6.96% compared to the reference group, so this study supports the classic work physiology notion that an enhanced cardiorespiratory fitness reduces the relative aerobic workload among workers exposed to high OPA [12,60]. Aerobic workload depends on cardiorespiratory fitness and RHR [9]. Thus, a reduced aerobic workload would be expected, based on a combination of an enhanced cardiorespiratory fitness and a reduced RHR. High aerobic workload strains the cardiovascular systems [61], which may potentially lead to progression of arteriosclerosis [15,62]. Thus, such a reduction in relative aerobic workload as observed in this study is considered to reduce the overall risk of CVD [14].

No changes were seen in either the resting or the ambulatory systolic and diastolic BP in the fully adjusted between groups analysis after 12-months. Likewise, previous worksite interventions with physical activity have not succeeded in changing the BP [54,63].

Between-group comparisons including all randomized participants show a 44.22% decrease in hsCRP in the aerobic exercise group compared to the reference group. Previous studies among healthy populations indicate that moderate-to-high-intensity leisure time aerobic exercise decreases the levels of hsCRP [64–67]. This might explain why this worksite aerobic exercise intervention reduced the level of hsCRP. A difference in hsCRP of this magnitude are estimated to lower the CVD risk by approximately 15% [68], and may therefore be considered clinically relevant. Thus, this aerobic exercise intervention seems to improve inflammatory levels among cleaners.

Between-group comparisons including all randomized participants show reductions (<1%) of body weight (Fig 4), body fat and waist circumference in the aerobic exercise group compared to the reference group. The magnitude of the reduction in waist circumference is estimated to lower the CVD risk by approximately 2% [69].

Overall this study showed significant increases of resting heart rate, hsCRP, fibrinogen and body weight at 12-months follow-up in the within group analysis among the reference group. However, in a RCT study as this, it is necessary to evaluate the between groups effects before and after the intervention for excluding effects on the outcome from other factors being common for both groups. For example, their physically demanding job and one year increased age may induce negative health effects on the entire study population. Thus, this is likely to lead to measured negative health in the reference group, but not in the aerobic exercise group which have sustained their health due to the aerobic exercise. Because aerobic exercise and reference groups were included from each respective workplace, we are not aware of any activities or factors which could have exclusively influenced the health of the reference group independently from the aerobic exercise group.

### Stratified analysis

No significant changes in resting BP were seen in the secondary between groups analysis stratified on the baseline level of aerobic workload among those exposed to an aerobic workload ≥30% HRR. However, the magnitude of the estimates of change in resting BP (9.95 mmHg systolic and 3.36 mmHg diastolic) may cause concern with respect to increasing the risk for CVD, due to the potential risk of overstraining the cardiovascular system as proposed previously [10,23]. Likewise, at the 4-months follow-up those participants exposed to high aerobic workloads showed an increase of 6.7 mmHg (p = 0.01) in systolic BP and an unaltered diastolic BP [10]. Although, the stratified results at the 12-months follow-up do not reach statistical significance the resting BP (9.95 mmHg (p = 0.08) in systolic BP and 3.36 mmHg (p = 0.21) in diastolic BP) still seems to have risen. The lack of significance may be owed to lack of power because the study was not designed for this stratification. Thus, the observed increased BP following 4-months of intervention among those participants exposed to high aerobic workloads seems to sustain after 12-months. Therefore, this is a potential adverse intervention effect which is strongly recommended to be further investigated.

### Methodological considerations

The convenience sampling of only three companies in the area of Copenhagen, Denmark limits the generalizability of the findings. Additionally, these findings are limited to cleaners. The fact that this worksite intervention was implemented among cleaners avoided socio economic confounding to the intervention. However, the external validity was enhanced by the creation of an aerobic exercise and reference group at each respective company. The study was sufficiently powered to detect clinically relevant changes. The cluster-randomized controlled trial design is a methodological strength reducing possible bias and contamination. Also the ITT analysis is a methodological strength reducing possible bias from differences in adherence [70]. The mixed model analysis is a strength when evaluating repeated measurements observations with observations missing at random, since the mixed model enables use of information from all observations without imputing missing observations [48,71]. The mixed-model analysis was applied, since it allows an ITT analyses with missing observations [71], which increased the n in the analysis due to some differences in missing data between groups, at baseline and follow-up.

In the sensitivity analysis excluding those using antihypertension and/or heart medication on a daily basis (n = 14) the majority of the obtained results were numerically and statistically similar to the results for the entire randomized population (Table 2). Additionally, the sensitivity analysis excluding participants reporting daily use of cholesterol and/or hormone medication (n = 12) showed results numerically and statistically similar to those for the randomized population (Table 2). Similar results both when including and excluding those participants using medication, which might have an impact on the results implies the roboustness of the results.

Altogether, almost one fourth of the study population was excluded from testing possibly leading to a differential selection bias towards better outcomes in a more healthy population. On the other hand, the larger benefits in terms of enhanced cardiorespiratory fitness appeared among those with medication for pre-existing hypertension or heart disease. Due to the rather small numbers of observations in the ABP measurements, these results may be considered supplementary to the results of the resting BP. However, the same directions of the estimates of change were confirmed by the ABP and using both measurements of resting and ambulatory BP strengthens the validity of the BP findings. It may be considered limitations that no attempt was done to provide the economical cost benefit or that the intensity of the aerobic exercise were not collected from 4 to 12-months follow-up.

### Practical implications

Intervention studies among cleaners evaluating the long term effect are scarce maybe because the group presents a number of well-known challenges for adherence and compliance. This emphasizes the importance of reporting all carefully collected data from the present study to inform future studies.

Cleaners suffer from an increased risk of CVD [7,8], that may partly be explained by a low cardiorespiratory fitness and high aerobic workload [24]. Previous research has concluded that the combination of a low cardiorespiratory fitness and high levels of OPA increase the risk of CVD [19,20]. In this study, the objective measurements revealed that more than half (51%) of the participants exceeded recommended levels of relative aerobic workload during working hours at baseline [10]. The effects of this aerobic exercise worksite intervention were significant reductions of the relative aerobic workload during working hours at 4-months [10] that was maintained in the 12-months follow-up despite less supervised aerobic exercise sessions. The previously reported adverse effects on 4-months follow-up on resting and ambulatory BP [10,23] were not present at 12-months follow-up among the randomized population (Table 2). However, the analysis stratified on relative aerobic workload at baseline revealed that those exposed to a high relative aerobic workload seems to obtain a notable adverse effect on resting and ambulatory BP. Hence, there is a need for more knowledge concerning the effects of aerobic exercise related to risk of CVD among occupational groups exposed to high OPA and aerobic workloads exceeding the international recommendations [72]. Hence, initiatives for safely reductions of aerobic workload and cardiovascular risk among high-risk occupational groups with high level of occupational physical activity are recommended [20,73].

## Conclusion

Based on a cluster-randomized design and objective diurnal data, this study showed significantly increased cardiorespiratory fitness, decreased RHR, aerobic workload and hsCRP. No changes between-groups were seen in SHR and BP both when measured at rest and during 24 hours. This indicates that the worksite aerobic exercise intervention among cleaners leads to several beneficial effects, which is likely to lower the risk for CVD.

Despite of these general positive effects, the increased resting BP of 10.0 mmHg systolic and 3.4 mmHg diastolic following the intervention among the cleaners exposed to a high relative aerobic workload may impose a significant adverse effect on CVD risk. Future studies are recommended to specifically investigare effects and physiological mechanisms of aerobic exercise worksite intervention among workers exposed to high OPA and relative aerobic workloads exceeding international recommendations.

## Supporting Information

S1 File CONSORT checklist. (DOC)

S2 File Protocol paper. (PDF)

S3 File Data set file. (SAV)

## Abbreviations

ABP: Ambulatory blood pressure
BMI: Body mass index
BP: Blood pressure
bpm: Beats per minute
CVD: Cardiovascular disease
HDL: High density lipoprotein
HR: Heart rate
HR_max_: Maximum heart rate
HRR: Heart rate reserve
% HRR: Percentage of heart rate reserve
hsCRP: High sensitive C-reactive protein
ITT: Intention to treat
LDL: Low density lipoprotein
LTPA: Leisure time physical activity
OPA: Occupational physical activity
RHR: Resting heart rate
SHR: Sleeping heart rate
VO_2_max: Maximum oxygen consumption
