# Supplementary material for: Long Term Effects on Risk Factors for Cardiovascular Disease after 12-Months of Aerobic Exercise Intervention - A Worksite RCT among Cleaners
Source: PLoS One. 2016 Aug 11;11(8):e0158547. doi: 10.1371/journal.pone.0158547 (PMC4981369; doi:10.1371/journal.pone.0158547)
Supplement: S2 File — (PDF) [file pone.0158547.s002.pdf]

Korshøj et al. BMC Public Health 2012, 12:645
http://www.biomedcentral.com/1471-2458/12/645

BMC Public Health

STUDY PROTOCOL **Open Access**

Cardiorespiratory fitness, cardiovascular workload and risk factors among cleaners; a cluster randomized worksite intervention

Mette Korshøj[1,2], Peter Krustrup[2,3], Marie Birk Jørgensen[1], Eva Prescott[5], Åse Marie Hansen[1,6], Jesper Kristiansen[1], Jørgen Henrik Skotte[1], Ole Steen Mortensen[1,7], Karen Søgaard[4] and Andreas Holtermann[1*]

Abstract

Background: Prevalence of cardiovascular risk factors is unevenly distributed among occupational groups. The working environment, as well as lifestyle and socioeconomic status contribute to the disparity and variation in prevalence of these risk factors. High physical work demands have been shown to increase the risk for cardiovascular disease and mortality, contrary to leisure time physical activity. High physical work demands in combination with a low cardiorespiratory fitness infer a high relative workload and an excessive risk for cardiovascular mortality. Therefore, the aim of this study is to examine whether a worksite aerobic exercise intervention will reduce the relative workload and cardiovascular risk factors by an increased cardiorespiratory fitness.

Methods/design: A cluster-randomized controlled trial is performed to evaluate the effect of the worksite aerobic exercise intervention on cardiorespiratory fitness and cardiovascular risk factors among cleaners. Cleaners are eligible if they are employed ≥ 20 hours/week, at one of the enrolled companies. In the randomization, strata are formed according to the manager the participant reports to. The clusters will be balanced on the following criteria: Geographical work location, gender, age and seniority. Cleaners are randomized to either I) a reference group, receiving lectures concerning healthy living, or II) an intervention group, performing worksite aerobic exercise "60 min per week". Data collection will be conducted at baseline, four months and 12 months after baseline, at the worksite during working hours. The data collection will consist of a questionnaire-based interview, physiological testing of health and capacity-related measures, and objective diurnal measures of heart rate, physical activity and blood pressure. Primary outcome is cardiorespiratory fitness.

Discussion: Information is lacking about whether an improved cardiorespiratory fitness will affect the cardiovascular health, and additionally decrease the objectively measured relative workload, in a population with high physical work demands. Previous intervention studies have lacked robust objective measurements of the relative workload and physical work demands. This study will monitor the relative workload and general physical activity before, during after the intervention, and contribute to the understanding of the previously observed opposing effects on cardiovascular health and mortality from occupational and leisure time physical activity.

Trial registration: The study is registered as ISRCTN86682076.

Keywords: Worksite intervention, Cleaners, Aerobic exercise, Work demands, Physical activity, Ambulatory blood pressure

* Correspondence: aho@nrcwe.dk
[1]National Research Centre for the Working Environment, Lersø Parkallé 105, 2100, Copenhagen Ø, Denmark
Full list of author information is available at the end of the article

BioMed Central

© 2012 Korshøj et al.; licensee BioMed Central Ltd. This is an Open Access article distributed under the terms of the Creative Commons Attribution License (http://creativecommons.org/licenses/by/2.0), which permits unrestricted use, distribution, and reproduction in any medium, provided the original work is properly cited.

Background

The prevalence of precursors of cardiovascular diseases such as hypertension and obesity is unevenly distributed across occupational groups and ethnicities [1-4]. This distribution in prevalence of cardiovascular risk factors reflects variation in lifestyle behaviour and socioeconomic status [1-4]. Factors within the working environment, as well as lifestyle behaviour and socioeconomic status contribute to the disparity and variation in the prevalence of these diseases between occupational groups [5-7]. Factors in the working environment receiving most attention in relation to cardiovascular diseases are psychosocial stressors, shift work and occupational noise [8]. More recently, high physical work demands have been shown to increase the risk of cardiovascular disease [9] and mortality [10]. This may be explained by upper limb activity increases heart rate and blood pressure [11] at the same absolute intensity as during lower limb activity. Additionally will the relatively higher aerobic workload for performing an absolute work task with low, compared to high, cardiorespiratory fitness, Figure 1, [12] will contribute to an excessive stress on the arterial wall and thereby it could constitute a cardiovascular risk. One potential explanation of this is the inflammation and cholesterol deposition in arterial endothelia, decreasing the lumen-diameter and the level of contractility of arterial layer of smooth muscle, giving rise eventually to arteriosclerosis and hypertension [13].

This proposed physiological mechanism is supported by the finding that a combination of high physical work demands and low cardiorespiratory fitness confers excessive risk for cardiovascular disease mortality [14,15], hypothetically by the increased relative aerobic workload, Figure 1. This may be explained by the higher relative aerobic workload and excessive wall-stress on arterial endothelia, experienced by an employee with low, compared to an employee with high, cardiorespiratory fitness [16].

In line with this, the international recommendations for aerobic workload are based on the physical work demands relative to the cardiorespiratory fitness of the employee [17-19]. The present international recommendation for aerobic workload is a maximum average intensity of 30 % of maximal oxygen consumption during an 8 hour working day [17-19].

Strategies to reduce the risk of cardiovascular disease in occupational groups with high physical work demands have among others included implementation of ergonomic adjustments for reducing the aerobic workload [20]. However, these adjustments have not been successful in reducing the aerobic workload [20,21]. Another strategy for reducing the relative aerobic workload is to implement initiatives aiming at improved cardiorespiratory fitness, [15,22]. In theory, an increased cardiorespiratory fitness will reduce the relative aerobic workload, Figure 1 [23]. However, high quality studies evaluating the effects on aerobic workload of intensive aerobic exercise and increased cardiorespiratory fitness are lacking [22].

Cleaners constitute an occupational group with high prevalence of hypertension, obesity and cardiovascular disease [7,24], which is in agreement with their well-documented high physical work demands and low cardiorespiratory fitness [12,25]. Several initiatives for decreasing the physical work demands among cleaners have been evaluated [23,25], but with no reports about significant effects on either the workload or cardiovascular health [7]. Furthermore, no previous study has investigated the effects on aerobic workload and cardiovascular risk factors following an intervention with intensive aerobic exercise, aiming at increased cardiorespiratory fitness among cleaners.

Therefore, the main aim of this study is to examine whether a worksite aerobic exercise intervention among cleaners will reduce the relative workload and cardiovascular risk factors by increasing cardiorespiratory fitness. The main hypotheses of the study are that the intervention will 1) increase cardiorespiratory fitness, and 2) decrease diurnal ambulatory blood pressure.

Methods and design

Study design

A cluster-randomized controlled trial will be conducted to evaluate the effect of a worksite intervention on cardiorespiratory fitness, relative aerobic workload and cardiovascular risk factors among cleaners. The cleaners consenting to participate in the trial will be randomized to either I) a reference group receiving 5 lectures about healthy living during the one year intervention period, or II) an intervention group performing worksite aerobic exercise of 60 minutes duration per week.

Outcomes will be measured at baseline, 4 months and 12 months after baseline. The study has been approved by the Danish data protection agency and the Ethics Committee for the regional capital in Denmark (journal

$$\text{Relative aerobic workload} = \frac{\text{Physical work demands}}{\text{Individual capacity (Cardiorespiratory fitness)}}$$

Figure 1 Conception of the relative workload.

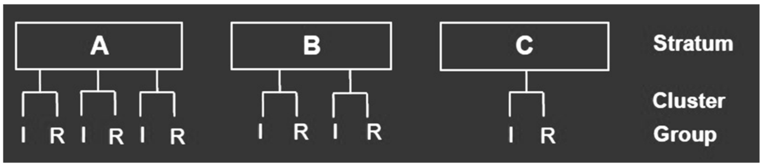

Figure 2 Randomization procedure, multilevel design. The clusters are randomised into either reference (R) or intervention group (I). Stratum (A, B, C) are defined by the manager the participant reports to. Clusters are formed from geographical work location, gender, age and seniority. Clusters are paired according to number of participants, gender, age and seniority.

number H-2-2011-116) and will be conducted in accordance with the Helsinki declaration. Prior to written consent to participate, information about the general aims of the study will be given to participants. The study is registered as ISRCTN86682076 in the current controlled trials.

The study is divided into two intervention phases with different aims. The aim of the first phase is to evaluate the efficacy of the intervention on cardiorespiratory fitness and cardiovascular risk factors. The aim of the second phase is to evaluate the effectiveness of the intervention on maintenance of coherence and cardiorespiratory fitness.

Power calculation

The power calculation shows that an expected increase of 4 % in cardiorespiratory fitness will take 52 participants in each of the two intervention groups to show significance at a level of 0.05 %. The expected increase in cardiorespiratory fitness is based on an expected drop out of participants of 30 %. It is planned to recruit 52 participants for each intervention group, to attain this participation, we plan to present the study for 130 cleaners, according to the assumption of a 40 % recruitment to the study.

Earlier studies show an increased cardiorespiratory fitness in physical activity interventions at the worksite of 7 – 10 % [26,27]. This study will be analyzed according to the intention to treat and therefore are the expected drop out included in the expected increasement of cardiorespiratory fitness. The expected drop out will decrease the average increased cardiorespiratory fitness, explained by the relative smaller increasement in cardiorespiratory fitness of the drop outs than the participants following the whole intervention.

Study population

The study population will consist of cleaners performing predominantly cleaning in day-care institutions, offices, hospitals and schools. The cleaners are employed at one of the enrolled companies in the suburban area of Copenhagen, Denmark.

Inclusion criteria at company level were more than 50 employed cleaners, possibility for the cleaners to participate in the project-activities during paid working time and location of the company in the suburban area of Copenhagen. Inclusion criteria at participant level were employment as cleaning assistant for > 20 hours per week, being between 18 and 65 years of age, and giving signed, informed consent.

Exclusion criteria for participating in the intervention are employment < 20 hours per week as a cleaning assistant, age below 18 years or above 65 years, declining to sign the informed consent, and pregnancy. Further, criteria of exclusion to specific physical tests are congestive heart failure, hospital admission for myocardial infarction or acute coronary syndrome within the last two years, hypertension (≥ 160/≥ 100 mmHg), angina pectoris, serious or chronic illness, severe trauma, frequent migraine, fever on the day of testing, and allergy to adhesive plasters for the diurnal measurements.

Recruitment of the study population

Recruitment of companies took place by direct contact by phone or email of management in cleaning companies in the suburban area of Copenhagen, Denmark. If the management showed an interest in taking part in the project, a meeting was arranged by the project leader and management staff of the company. At the meeting, the aim, content and activities of the project were described and the possibility of enrolment to the study was discussed. Once collaboration was confirmed, details about the recruitment of employees were settled. All employees were invited to an information meeting where the project was described, and assignment to voluntarily participation was conducted via a screening questionnaire. Prior to the information meeting, written information about the aim, content and activities was distributed to all employees.

Randomization

The employees who volunteer to participate in the study and attend the baseline health check will be randomized to either a reference or an intervention group. The randomization will be performed at cluster-level for prevention of contamination between participating colleagues, Figure 2. Clusters are set within strata. Each stratum is formed according to which manager the

participant reports to. The clusters will be balanced on geographical work location, gender, age and seniority. Within each stratum, the clusters will be paired to minimize imbalance across several strata. Clusters will be paired according to number of participants, gender, age and seniority. Strata will be named alphabetically, and clusters will be named consecutively within each stratum. The randomization will be carried out by researchers blinded to the identity of the strata and clusters. All paired clusters assigned to the specific stratum will be drawn from an opaque, tossed bag and will be alternately allocated to either reference or intervention group, depending of the flip of a coin. Tails will decide allocation of the first of the two drawn paired clusters to the reference group and heads to the intervention group. The second of the two drawn paired clusters will be allocated to the opposite group to the first.

Development and planning of the intervention

The activities offered to the intervention group are specified and adjusted to the respective company by a modified intervention mapping approach [28]. The intervention mapping facilitates participation and consultation of all participating stakeholders (organization, management, employees, researchers, intervention- and test-instructors).

The detailed protocol for the intervention was based on three key points: 1) feasibility; it should be possible to execute the intervention at or nearby the enrolled company, during paid working time; 2) motivation; the intervention activities should apply to the participants' preferences; 3) a standardized protocol for a scientific evaluation. According to the steps of the intervention mapping [28], the following were defined, Table 1.

Reference group

The reference group receives 5 lectures of 2 hours/lecture during the one year intervention period. The lectures will concern healthy living. None of the lectures will address physical activity. Participants in the reference group will be invited to give suggestions for the proposed lectures. The lectures will be held at the enrolled company during paid working time.

Intervention group

The intervention group will be offered supervised aerobic exercise of 60 minutes duration split into 2–3 weekly sessions during the first phase of the intervention. The weekly number of sessions will be decided within the individual company. The degree of supervision will be reduced during the second phase of the intervention, in which the participants will be encouraged to continue with the aerobic exercise themselves. The progressive reduction in supervision is to investigate the ability of the cleaners to participate and perform the aerobic exercise without external involvement.

To increase the cardiorespiratory fitness, the aerobic exercise will be aimed to be performed at an intensity of >60 % of maximal oxygen consumption (VO_2max) [29]. The types of aerobic exercise will be tailored to the specific worksite through a modified intervention mapping approach, Table 1.

Data collection and study materials

Data collection will be conducted at three time points: At baseline, four and 12 months after baseline. The data collection will consist of a health check consisting of a questionnaire-based interview, physical testing of health and capacity-related measures, and objective diurnal measures of heart rate, physical activity, body position and blood pressure. At all time points, the questionnaire and the data collection will be conducted by test instructors blinded to the randomization of the participants.

The participant will get instant feedback from the physical testing in the health check, except for the venous blood sample, from a test-instructor. If some of the measured values are above the recommended levels from international and Danish health organizations [30,31], the participant will be informed and encouraged to contact a physician.

A structured interview with validated measures will be conducted. The interview will involve sociodemographic measures i.e. sex, ethnicity, country of birth, occupational group, employment status and education [32], lifestyle behaviour and health i.e. alcohol consumption, medicine use, parental history of cardiovascular disease, sleeping behaviour, level of physical activity in occupation and leisure [33], general health, modified items of the Nordic questionnaires for the analysis of musculoskeletal disorders [34], rate of perceived physical exertion [35], items from Copenhagen psychosocial Questionnaire [36], and items from the workability scale [37].

Objective physical measures of weight, percent body fat, (Tanita BC418), height (seca model 213 1721009), body mass index (BMI), waist and hip circumference (WHR) (seca 201), a venous blood sample, blood pressure (Omron M6 comfort) and level of cardiorespiratory fitness by a sub-maximal step test.

Weight and percent body fat will be estimated by a Tanita BC418. Measurement will be made while the participant is wearing light clothes and no shoes. Estimated weight of clothes (1.5 kg) will be subtracted from body weight. Percent body fat will be estimated by bio-electric-impedance-analysis from electricity running from foot to foot and hand to hand [38]. Height will be measured while the participant is standing looking straight forward and wearing no shoes. Calculation of BMI by the equation of BMI = (body weight (kg)/body

Table 1 The plan and overview of the intervention mapping procedure

Outcomes	Tasks	
Programme objectives	*Performance objective*	- decrease risk of cardiovascular disease
	Changeable determinant	- increase cardiorespiratory fitness
	Target population	- cleaners
Theoretical methods	*Literature review*	- > 60 % of VO_2max > 60 min/week
	Method to strategy	- worksite-adjusted intervention
Programme design	*Strategy to plan*	- facilitating specific worksite group
	Instruction materials	- aerobic exercise sessions
	Pre-test materials	- pilot study with cleaners
	Intervention materials	- adjusted aerobic exercise sessions
Adoption and implementation plan	*Linkage system*	- facilitating specific worksite group
	Adoption objectives	- registration of conducted planned activities
		- target group participation
	Adoption determinants	- logistic planning of work and activities
		- collaboration between organisation and researchers
	Implementation plan	- activities planned in collaboration
Monitoring and evaluation plan	*Evaluation model*	- participation registration
		- on-going adjustments in aerobic exercise
	Effect evaluation	- increased cardiorespiratory fitness
		- decreased diurnal blood pressure
		- improve self-rated work productivity
		- reduce rate of RPE during work
		- improve metabolic cardiovascular risk factors
		- reduce the need for recovery
		- reach of target group
	Process evaluation	- delivering of activities
		- receipt of activities

height (m^2)) [30,39]. Waist and hip circumference will be measured while the participant wears light clothes and is standing looking straight forward. The waist is defined as the narrowest point between the lowest rib and the iliac crest [30,39]. The WHR is calculated by waist circumference (cm)/hip circumference (cm). A 25 ml blood sample will be drawn from the vena brachialis by a bio-analyst. Blood pressure will be measured on the left upper arm after 15 minutes of sitting at rest. Level of cardiorespiratory fitness will be estimated by a sub-maximal step test. The step test is conducted on a bench of 30 cm height for females and 35 cm for males. The step frequency is increased from 0.2 steps per second to maximal 0.8 steps per second, at the maximal 6 minutes of testing time. The step test is terminated when the participant is unable to follow the rhythm of the steps or unable to extend the knee properly.

Diurnal measurements of heart rate, physical activity, body position and ambulatory blood pressure will be conducted after each health check. Heart rate, physical activity and body position will be measured over four days and during both working and non-working days. Ambulatory blood pressure will be measured over 24 hours on a day including work. Cleaners will be instructed to write a log of working hours, sleeping and waking time and time periods spent without monitors. Cleaners will be given practical information about how to wear and treat the monitors.

The diurnal measurements of heart rate will be performed with Actiheart [40], to assess type of physical activity and body position the Actigraph GT3X + [41] will be used and for ambulatory blood pressure the Spacelabs 90217 [42].

Actiheart measures electrocardiographic raw signals with a sensitivity of 0.25 mV. Heart rate is calculated by the interbeat intervals between the R peaks in the QRS complex. Actiheart is initialised by the height, weight, gender and age of each participant. Actiheart is validated for measurement of heart rate, heart rate variability and

estimation of energy expenditure in the field [43-45]. Actiheart will be mounted with ag-ag-cl pre-gelled electrodes (Ambu blue sensor VL-00-S/25) at one of the validated positions at the apex of sternum with a horizontal wire to the right at the level of the 5th and 6th intercostal space or at the manubrium of the sternum with a horizontal wire to the right at the level of the 2th and 3th intercostal space [46].

Actigraph measures accelerations in 3 dimensions with a dynamic range of ± 6 G, sampled with a precision of 12 bit. Actigraph is validated for estimation of type of physical activity in the field [47,48]. The accelerometers are initialised for recording and data downloaded using the manufacturer's software (ActiLife version 5.5). Four Actigraphs will be mounted on the skin with adhesive tape (3 M, Hair-Set, double sided adhesive tape and Fixomull, BSN medical) at the level of T1-T2, 3 cm distal to the deltoid insertion on the dominant arm, laterally to and below the iliac crest [48] and on the right thigh at the most muscular part of the quadriceps femoris, medial to the iliac crest and the top of the patella, orientated with the x-axis pointing downwards, y-axis horizontally to the left and z-axis horizontally forward. The Actigraph signals will be sampled at 30 Hz, and processed to derive the amount of steps, type of physical activity (e.g. walking, climbing stairs, running, arms above shoulder height, bending of the spine), and body positions (e.g. standing, sitting and lying) [45].

Spacelabs measures ambulatory blood pressure by oscillometry. Spacelabs is validated for measurement of blood pressure in the field [49]. The Spacelab monitor will be mounted on the non-dominant upper arm with a tube connecting the sampler to the cuff. The sampler is mounted with elastic straps around the waist. Spacelabs will be used to monitor the ambulatory blood pressure over 24 hours, with a frequency of every 20 minutes during waking hours, and measurements every 40 minutes during sleep [50,51].

Outcomes

The primary outcome of this trial is cardiorespiratory fitness (mlO_2* min^{-1} * kg^{-1}), Figure 3. Secondary outcomes are diurnal heart rate, physical activity, body position and ambulatory blood pressure, relative aerobic workload (percent of heart rate reserve (%HRR)) self-rated work productivity, rate of perceived exertion (RPE) during work, intensity and number of regions with self-rated musculoskeletal pain, high sensitive C-reactive protein (hsCRP), high density lipoprotein (HDL), glycated haemoglobin (HbA1c), self-rated need for recovery and participation in intervention.

Outcomes will be measured before the randomization at baseline, as well as 4 and 12 months after baseline.

Analyses

From the diurnal measurements the following analyses will be made; 1) the relative aerobic workload is calculated by the heart rate reserve (HRR), by the difference between the estimated maximal heart rate (HR_{max}) [52] and the sleeping heart rate, defined as the 10th lowest recorded heart rate value during sleep (SHR) [53] ($HRR = HR_{max} - SHR$). The HRR is similar to the percentage of VO_2max during whole body physical activity at group level [19,52], 2) the monitored diurnal blood pressure over 24 hours, will be analyzed according to the guidelines for ambulatory blood pressure [49], 3) the body position, level of physical activity and amount of steps and step frequency will be estimated [48,54].

The health check data will be analyzed according to the listed outcomes; 1) cardiorespiratory fitness is estimated based on the step frequency and stepping time obtained at the sub maximal step test and qualified as poor, average or good [55], 2) relative aerobic workload (% HRR), diurnal blood pressure and level of hsCRP [56] and HDL [57]. All data will be analyzed based on the intention-to-treat population, and as a per protocol analysis based on level of participation in the intervention activities.

Statistical analysis

The effect of the primary and secondary outcomes will be analyzed by multilevel analyses. The analyses will be performed after the three time points of measurement: baseline, after 4 months (short term) and after 12 months (long term).

Discussion

Only sparse information exists about how an intervention increasing cardiorespiratory fitness will affect the objectively measured relative aerobic workload as well as

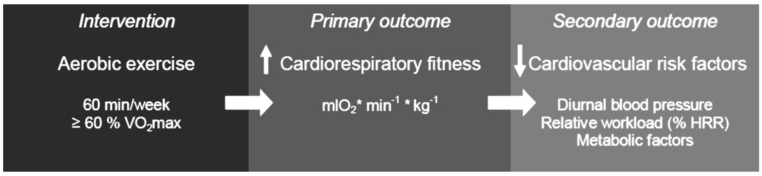

Figure 3 Conceptual model of the project. The model describes the intervention, the primary and the secondary outcomes.

cardiovascular risk factors, in a population with high physical work demands. Therefore, the main aim of this study is to examine whether a worksite aerobic exercise intervention will reduce the relative aerobic workload and cardiovascular risk factors by an increased cardiorespiratory fitness among cleaners.

It is well known that aerobic exercise is highly effective in increasing cardiorespiratory fitness and reducing cardiovascular risk factors [58,59]. However, this study will focus on the implementation of physical activity and the effect in terms of reduction in relative aerobic workload and cardiovascular risk factors in a working population with high physical work demands. Earlier intervention studies in this field have lacked robust, objective measurements of the relative aerobic workload, occupational physical activity and physical work demands in general. This study will provide objective data on the relative aerobic workload and general physical activity before, during and after the worksite aerobic exercise intervention. In addition, cardiovascular risk factors such as diurnal blood pressure will be monitored and evaluated.

Impact of results

Information from effect and implementation of the intervention may be beneficial for future reduction and prevention of cardiovascular risk factors in blue collar workers. Blue collar workers are well known to have an increased prevalence of health issues like cardiovascular disease [4,5,7,60], obesity, hypertension and musculoskeletal disorders [61]. Knowledge about implementation of health initiatives like physical activity for this work group is therefore particularly important.

Moreover, existing knowledge about effects of physical activity on cardiovascular risk is mainly based on male populations [58,59,62]. The cleaning sector in Denmark primarily employs women, and this study will therefore enhance the scientific knowledge about effects of physical activity on cardiovascular risk factors among women.

The impact of enhanced cardiorespiratory fitness on the relative aerobic workload will be measured with the objectively recording systems, providing objective diurnal information over several days regarding percentage of HRR and diurnal blood pressure. Diurnal objective measures of physical activity, such as body position, will be measured by triaxial accelerometry. Occupational workload has previously been objectively measured among cleaners, public administrators and other occupational groups [25,50,63], but none of these studies have simultaneously measured body position, physical activity and the aerobic workload. Simultaneous, objective collection of heart rate, blood pressure, body position and physical activity will enable more detailed information about the occupational workload and the related cardiovascular responses among cleaners to be included in the activity profile. To our knowledge, such a detailed analysis has not previously been conducted during free living subjects for several days.

The impact of the intervention will also be analyzed including several factors known to increase the cardiovascular risk, i.e. the metabolic factors HDL and hsCRP. Measurement of metabolic factors offers the possibility of a comprehensive in depth analysis not previous seen for a field intervention study among cleaners and other blue collar populations.

Besides the physiological effects on relative aerobic workload and cardiovascular health, the intervention may also have a positive impact on the subjective feeling of strain and physical work exhaustion during [35] and after work as well as need for recovery, previously shown to be related to cardiorespiratory fitness [64-66].

In addition, as this study combines diurnal objective measurements of physical activity over several days, with an physical exercise intervention, it will contribute to our understanding of previously observed contradictory effects on cardiovascular risk factors and mortality of work and leisure time physical activity [9,10,67].

Strengths and limitations of the study

A strength of the study is the diurnal objective measurement of physical activity, body positions, heart rate and blood pressure over several days, which will decrease the risk of subjective recall bias, and bias from factors such as individual physical capacity and symptoms. The cluster-randomized controlled trial design is a methodological strength, since it minimizes the risk of contamination between the intervention and reference group, and reduces the risk for bias.

The systematic intervention mapping approach is a strong feature as the experience and information obtained in the process of implementing an exercise initiative in a cleaning company will be captured and benefit the project and future studies. Another strength of the study is that the project takes place at several companies, with reference and intervention groups within each company, reducing the risk of uneven distribution of participants in the project as a whole, should a company withdraw from the project, this greatly enhance the external validity.

A limitation is the restriction of recruitment to companies in the area of Copenhagen. The companies and participants may therefore not be representative of cleaning companies and cleaners in Denmark as a whole, and therefore limit the generalizability of the findings.

In summary, this study will investigate the effect of a worksite intervention aiming to enhance the cardiorespiratory fitness, and reduce aerobic workload and cardiovascular risk factors, in an occupational group with a high physical work demands and generally poor cardiovascular

health. The intervention will be evaluated on the basis of objective diurnal measurements of, amongst other factors, heart rate, body position and blood pressure. Additionally, the impact of the intervention on established cardiovascular risk factors will be evaluated. The intervention will be developed in collaboration with the participating companies, and will contribute information about the effects of introducing an aerobic exercise intervention in an occupational group greatly in need of health promoting initiatives.

Abbreviations
BMI: Body mass index; HbA1c: Glycated haemoglobin; HDL: High density lipoprotein; HRmax: Maximal heart rate; HRR: Heart rate reserve; hsCRP: High sensitive C-reactive protein; RPE: Rate of perceived extersion; SHR: Sleeping heart rate; VO_2max: Maximal oxygen consumption; WHR: Waist to hip ratio.

Competing interests
The authors declare that they have no competing interests.

Authors' contributions
MK, PK, MBJ, KS and AH participated in the discussion of the conceptual design of the study and wrote the initial protocol as well as the design of the intervention. MK was responsible for drafting the paper, writing the trial registration and application for the ethical committee. EP, ÅMH, JK, JS, and OSM supervised the discussion and choice of methods for measurements and analysis. All authors have read and commented on the draft version as well as approved the final version of the manuscript.

Acknowledgements
The study is externally funded by The Danish Working Environment Research Fund (journal number 20100019624/5) with 1,8 mio dk.kr.

Author details
[1]National Research Centre for the Working Environment, Lersø Parkallé 105, 2100, Copenhagen Ø, Denmark. [2]Department of Exercise and Sport Sciences, Section of Human Physiology, University of Copenhagen, Nørre Allé 51, 2200, Copenhagen, N Denmark. [3]Sport and Health Sciences, College of Life and Environmental Sciences, St. Luke's Campus, University of Exeter, Exeter, UK. [4]Institute of Sports Science and Clinical Biomechanics, University of Southern Denmark, Campusvej 55, 5230, Odense M, Denmark. [5]Department of Cardiology, Bispebjerg University Hospital, Bispebjerg Bakke 23 2400, Copenhagen, NV, Denmark. [6]Department of Public Health, University of Copenhagen, Øster Farimagsgade 5, 1014, Copenhagen K, Denmark. [7]Bispebjerg University Hospital, Bispebjerg Bakke 23, 2400, Copenhagen, NV, Denmark.

Received: 21 June 2012 Accepted: 7 August 2012
Published: 13 August 2012

References
1. Kurian AK, Cardarelli KM: **Racial and ethnic differences in cardiovascular disease risk factors: a systematic review.** *Ethn Dis* 2007, **17**:143–152.
2. Agyemang C, Addo J, Bhopal R, Aikins AG, Stronks K: **Cardiovascular disease, diabetes and established risk factors among populations of sub-Saharan African descent in Europe: a literature review.** *Global Health* 2009, **5**:7.
3. Gadd M, Johansson SE, Sundquist J, Wandell P: **Are there differences in all-cause and coronary heart disease mortality between immigrants in Sweden and in their country of birth? A follow-up study of total populations.** *BMC Public Health* 2006, **6**:102.
4. Marmot M: **Social determinants of health inequalities.** *Lancet* 2005, **365**:1099–1104.
5. Strand BH, Tverdal A: **Can cardiovascular risk factors and lifestyle explain the educational inequalities in mortality from ischaemic heart disease and from other heart diseases? 26 year follow up of 50,000 Norwegian men and women.** *J Epidemiol Community Health* 2004, **58**:705–709.
6. Stringhini S, Sabia S, Shipley M, Brunner E, Nabi H, Kivimaki M, Singh-Manoux A: **Association of socioeconomic position with health behaviors and mortality.** *JAMA* 2010, **303**:1159–1166.
7. Sjögren B, Fredlund P, Lundberg I, Weiner J: **Ischemic heart disease in female cleaners.** *Int J Occup Environ Health* 2003, **9**:134–137.
8. Virkkunen H, Harma M, Kauppinen T, Tenkanen L: **Shift work, occupational noise and physical workload with ensuing development of blood pressure and their joint effect on the risk of coronary heart disease.** *Scand J Work Environ Health* 2007, **33**:425–434.
9. Krause N, Brand RJ, Kaplan GA, Kauhanen J, Malla S, Tuomainen TP, Salonen JT: **Occupational physical activity, energy expenditure and 11-year progression of carotid atherosclerosis.** *Scand J Work Environ Health* 2007, **33**:405–424.
10. Holtermann A, Mortensen OS, Burr H, Søgaard K, Gyntelberg F, Suadicani P: **The interplay between physical activity at work and during leisure time - risk of ischemic heart and all-cause mortality in middle-aged Caucasian men.** *Scand J Work Environ Health* 2009, **35**:466–474.
11. Åstrand I, Guharay A, Wahren J: **Circulatory Responses to Arm Exercise with Different Arm Positions.** *J Appl Physiol* 1968, **25**:528–532.
12. Søgaard K, Blangsted AK, Herod A, Finsen L: **Work design and the labouring body: examining the impacts of work organization on Danish cleaners' health.** *Antipode* 2006, **38**:579–602.
13. Glagov S, Zarins C, Giddens DP, Ku DN: **Hemodynamics and Atherosclerosis - Insights and Perspectives Gained from Studies of Human Arteries.** *Arch Pathol Lab Med* 1988, **112**:1018–1031.
14. Holtermann A, Hansen JV, Burr H, Sogaard K, Sjogaard G: **The health paradox of occupational and leisure-time physical activity.** *Br J Sports Med* 2011, **46**:291–295.
15. Holtermann A, Mortensen OS, Burr H, Søgaard K, Gyntelberg F, Suadicani P: **Physical demands at work, physical fitness, and 30-year ischaemic heart disease and all-cause mortality in the Copenhagen Male Study.** *Scand J Work Environ Health* 2010, **36**:357–365.
16. Louhevaara V: **Job Demands and Physical Fitness.** In *The occupational ergonomics handbook*. Edited by Waldemar K.: William S. Marras. CRC Press; 1999:261–273.
17. Ilmarinen J: **Physical Load on the Cardiovascular-System in Different Work Tasks.** *Scand J Work Environ Health* 1984, **10**:403–408.
18. Jorgensen K: **Permissible Loads Based on Energy-Expenditure Measurements.** *Ergonomics* 1985, **28**:365–369.
19. Wu HC, Wang MJJ: **Relationship between maximum acceptable work time and physical workload.** *Ergonomics* 2002, **45**:280–289.
20. Søgaard K, Laursen B, Jensen BR, Sjøgaard G: **Dynamic loads on the upper extremities during two different floor cleaning methods.** *Clin Biomech* 2001, **16**:866–879.
21. Woods V, Buckle P: **Musculoskeletal ill health amongst cleaners and recommendations for work organisational change.** *Int J Ind Erg* 2006, **36**:61–72.
22. Proper KI, Koning M, Van der Beek AJ, Hildebrandt VH, Bosscher RJ, Van Mechelen W: **The effectiveness of worksite physical activity programs on physical activity, physical fitness, and health.** *Clin J Sport Med* 2003, **13**:106–117.
23. Sogaard K, Fallentin N, Nielsen J: **Work load during floor cleaning.** *The effect of cleaning methods and work technique. Eur J Appl Physiol Occup Physiol* 1996, **73**:73–81.
24. Zock JP: **World at work: Cleaners.** *Occup Environ Med* 2005, **62**:581–584.
25. Unge J, Ohlsson K, Nordander C, Hansson GA, Skerfving S, Balogh I: **Differences in physical workload, psychosocial factors and musculoskeletal disorders between two groups of female hospital cleaners with two diverse organizational models.** *Int Arch Occup Environ Health* 2007, **81**:209–220.
26. Dishman RK, Oldenburg B, O'Neal H, Shephard RJ: **Worksite physical activity interventions.** *Am J Prev Med* 1998, **15**:344–361.
27. Conn VS, Hafdahl AR, Cooper PS, Brown LM, Lusk SL: **Meta-analysis of workplace physical activity interventions.** *Am J Prev Med* 2009, **37**:330–339.
28. Bartholomew LK, Parcel GS, Kok G: **Intervention mapping: a process for developing theory- and evidence-based health education programs.** *Health Educ Behav* 1998, **25**:545–563.
29. Davies CTM, Knibbs AV: **The training stimulus. The effects of intensity, duration and frequency of effort on maximum aerobic power output.** *Int Z angew Physiol Arbeitsphysiol* 1971, **29**:299–305.
30. Canoy D: **Distribution of body fat and risk of coronary heart disease in men and women.** *Curr Opin Cardiol* 2008, **23**:591–598.
31. The WHO euro website: www.euro.who.int/en/home.

32. Holtermann A, Jorgensen MB, Gram B, Christensen JR, Faber A, Overgaard K, Ektor-Andersen J, Mortensen OS, Sjogaard G, Sogaard K: **Worksite interventions for preventing physical deterioation among employees in job-groups with high physical work demands: Background, design and conceptual model of FINALE.** *BMC Public Health* 2010, **9**:(10)120.
33. Saltin B, Grimby G: **Physiological analysis of middle-aged and old former athletes. Comparison with still active athletes of the same ages.** *Circulation* 1968, **38**:1104–1115.
34. Kuorinka I, Jonsson B, Kilbom Å, Vinterberg H, Biering-Sørensen F, Andersson G, Jørgensen K: **Standardised Nordic questionnaires for the analysis of musculoskeletal symptoms.** *Appl Ergo* 1987, **18**:233–237.
35. Borg GAV: *Physical performance and perceived exertion*. Lund: CWK Glerup; 1962.
36. Fallentin N, Juul-Kristensen B, Mikkelsen S, Andersen JH, Bonde JP, Frost P, Endahl L: **Physical exposure assessment in monotonous repetitive work–the PRIM study.** *Scand J Work Environ Health* 2001, **27**:21–29.
37. Ilmarinen J, Tuomi K, Klockars M: **Changes in the work ability of active employees over an 11-year period.** *Scand J Work Environ Health* 1997, **23**(Suppl 1):49–57.
38. *The Tanita web site*. www.tanita.com.
39. Han TS, Sattar N, Lean M: **ABC of obesity, Assessment of obesity and its clinical implications.** *British J Sports Med* 2006, **333**:695–698.
40. *The Camntech web site*. www.camntech.com.
41. *The Actigraph web site*. http://www.theactigraph.com.
42. *The Spacelabs Healthcare web site*. www.spacelabshealthcare.com.
43. Barreira TV, Kang M, Caputo JL, Farley RS, Renfrow MS: **Validation of the Actiheart Monitor for the Measurement of Physical Activity.** *International Journal Exercise Science* 2009, **2**(1):60–71.
44. Assah FK, Ekelund U, Brage S, Wright A, Mbanya JC, Wareham NJ: **Accuracy and validity of a combined heart rate and motion sensor for the measurement of free-living physical activity energy expenditure in adults in Cameroon.** *Int J Epidemiol* 2011, **40**(1):112–20. 011 Feb.
45. Kristiansen J, Korshoj M, Skotte JH, Jespersen T, Sogaard K, Mortensen OS, Holtermann A: **Comparison of two systems for long-term heart rate variability monitoring in free-living conditions - a pilot study.** *Biomed Eng Online* 2011, **12**(10):27.
46. Brage S, Brage N, Franks PW, Ekelund U, Wareham NJ: **Reliability and validity of the combined heart rate and movement sensor Actiheart.** *Eur J Clin Nutr* 2005, **59**:561–570.
47. Hart TL, McClain JJ, Tudor-Locke C: **Controlled and free-living evaluation of objective measures of sedentary and active behaviors.** *J Phys Act Health* 2011, **8**:848–857.
48. de Vries SI, Garre FG, Engbers LH, Hildebrandt VH, van Buuren S: **Evaluation of Neural Networks to Identify Types of Activity Using Accelerometers.** *Medicine and Science in Sports and Exercise* 2011, **43**:101–107.
49. Baumgart P, Kamp J: **Accuracy of the SpaceLabs Medical 90217 ambulatory blood pressure monitor.** *Devices and Technology* 1998, **3**:303–307.
50. Clays E, Leynen F, De Bacquer D, Kornitzer M, Kittel F, Karasek R, De Backer G: **High job strain and ambulatory blood pressure in middle-aged men and women from the Belgian Job Stress Study.** *J Occup Environ Med* 2007, **49**:360–367.
51. Clays E, Van HK, De BM, Kornitzer M, Kittel F, De BG, De BD: **Behavioural and psychosocial correlates of nondipping blood pressure pattern among middle-aged men and women at work.** *J Hum Hypertens* 2012, **26**:381–387.
52. Tanaka H, Monahan KD, Seals DR: **Age-predicted maximal heart rate revisited.** *J Am Coll Cardiol* 2001, **37**:153–156.
53. Brage S, Brage N, Franks PW, Ekelund U, Wong MY, Andersen LB, Froberg K, Wareham NJ: **Branched equation modeling of simultaneous accelerometry and heart rate monitoring improves estimate of directly measured physical activity energy expenditure.** *J Appl Physiol* 2004, **96**:343–351.
54. Skotte JH, Korshoj M, Kristiansen J, Hanisch C, Holtermann A: **Detection of physical activity types using triaxial accelerometers.** *J Physical Activity & Health* 2012 In press.
55. Shvartz E, Reibold RC: **Aerobic Fitness Norms for Males and Females Aged 6 Years to 75 Years - A Review.** *Aviat Space Environ Med* 1990, **61**:3–11.
56. Plaisance EP, Grandjean PW: **Physical activity and high-sensitivity C-reactive protein.** *Sports Med* 2006, **36**:443–458.
57. Cornelissen VA, Fagard RH: **Effects of endurance training on blood pressure, blood pressure-regulating mechanisms, and cardiovascular risk factors.** *Hypertension* 2005, **46**:667–675.
58. Kodama S, Saito K, Tanaka S, Maki M, Yachi Y, Asumi M, Sugawara A, Totsuka K, Shimano H, Ohashi Y, *et al*: **Cardiorespiratory fitness as a quantitative predictor of all-cause mortality and cardiovascular events in healthy men and women: a meta-analysis.** *JAMA* 2009, **301**:2024–2035.
59. Aspenes ST, Nilsen TI, Skaug EA, Bertheussen GF, Ellingsen O, Vatten L, Wisloff U: **Peak oxygen uptake and cardiovascular risk factors in 4631 healthy women and men.** *Med Sci Sports Exerc* 2011, **43**:1465–1473.
60. Mackenbach JP, Stirbu I, Roskam AJ, Schaap MM, Menvielle G, Leinsalu M, Kunst AE: **Socioeconomic inequalities in health in 22 European countries.** *N Engl J Med* 2008, **358**:2468–2481.
61. Jorgensen MB, Rasmussen CDN, Carneiro IG, Flyvholm MA, Olesen K, Ekner D, Sogaard K, Holtermann A: **Health disparities between immigrant and Danish cleaners.** *Int Arch Occup Environ Health* 2011, 1–10.
62. Hooker SP, Sui X, Colabianchi N, Vena J, Laditka J, LaMonte MJ, Blair SN: **Cardiorespiratory fitness as a predictor of fatal and nonfatal stroke in asymptomatic women and men.** *Stroke* 2008, **39**:2950–2957.
63. Halbert JA, Silagy CA, Finucane P, Withers RT, Hamdorf PA, Andrews GR: **The effectiveness of exercise training in lowering blood pressure: a meta-analysis of randomised controlled trials of 4 weeks or longer.** *J Hum Hypertens* 1997, **11**:641–649.
64. Robroek SJW, van de Vathorst S, Hilhorst MT, Burdorf A: **Moral issues in workplace health promotion.** *Int Arch Occup Environ Health* 2012, **85**:327–331.
65. Pronk NP, Martinson B, Kessler RC, Beck AL, Simon GE, Wang P: **The association between work performance and physical activity, cardiorespiratory fitness, and obesity.** *J Occup Environ Med* 2004, **46**:19–25.
66. de Croon EM, Sluiter JK, Frings-Dresen MH: **Psychometric properties of the Need for Recovery after work scale: test-retest reliability and sensitivity to detect change.** *Occup Environ Med* 2006, **63**:202–206.
67. Holtermann A, Mortensen OS, Sogaard K, Gyntelberg F, Suadicani P: **Risk factors for ischaemic heart disease mortality among men with different occupational physical demands. A 30-year prospective cohort study.** *BMJ Open* 2012, **2**:e000279.

doi:10.1186/1471-2458-12-645
Cite this article as: Korshøj *et al.*: Cardiorespiratory fitness, cardiovascular workload and risk factors among cleaners; a cluster randomized worksite intervention. *BMC Public Health* 2012 **12**:645.

Submit your next manuscript to BioMed Central and take full advantage of:

- **Convenient online submission**
- **Thorough peer review**
- **No space constraints or color figure charges**
- **Immediate publication on acceptance**
- **Inclusion in PubMed, CAS, Scopus and Google Scholar**
- **Research which is freely available for redistribution**

Submit your manuscript at
www.biomedcentral.com/submit

BioMed Central
